# Anti-MAdCAM Antibody Increases ß7+ T Cells and CCR9 Gene Expression in the Peripheral Blood of Patients With Crohn’s Disease

**DOI:** 10.1093/ecco-jcc/jjx121

**Published:** 2017-09-07

**Authors:** Mina Hassan-Zahraee, Anindita Banerjee, John B Cheng, Weidong Zhang, Alaa Ahmad, Karen Page, David von Schack, Baohong Zhang, Steven W Martin, Satyaprakash Nayak, Padma Reddy, Li Xi, Hendrik Neubert, Mireia Fernandez Ocana, Ken Gorelick, Robert Clare, Michael Vincent, Fabio Cataldi, Kenneth Hung

**Affiliations:** 1 Pfizer Inc., Cambridge, MA, USA; 2 Pfizer Inc., Andover, MA, USA

**Keywords:** Crohn’s disease, MAdCAM, PF-00547659, pharmacodynamics, treatment

## Abstract

**Objective:**

To define pharmacodynamic biomarkers in the peripheral blood of patients with Crohn’s disease [CD] after treatment with PF-00547659, an anti-human mucosal addressin cell adhesion molecule-1 [MAdCAM-1] monoclonal antibody.

**Methods:**

In this Phase 2, randomised, double-blind, controlled study [OPERA], blood samples were analysed from patients with moderate to severe active CD who received placebo or 22.5 mg, 75 mg, or 225 mg of PF-00547659 subcutaneously at baseline and at Weeks 4 and 8, with follow-up at Week 12. Soluble MAdCAM [sMAdCAM] was measured by mass spectrometry, β7-expressing T cells by flow cytometry, and gene transcriptome by RNA sequencing.

**Results:**

A slight increase in sMAdCAM was measured in the placebo group from baseline to Week 12 [6%], compared with significant decreases in all PF-00547659 groups [–87% to –98%]. A slight increase from baseline to Week 12 was observed in frequency and molecules of equivalent soluble fluorochrome for β7+ central memory T cells in the placebo group [4%], versus statistically significant increases in the active treatment groups [48% to 81%]. Similar trends were seen for β7+ effector memory T cells [placebo, 8%; PF-00547659, 84–138%] and β7+ naïve T cells [8%; 13–50%]. CCR9 gene expression had statistically significant up-regulation [*p* = 1.09e-06; false discovery rate < 0.1] with PF-00547659 treatment, and was associated with an increase in β7+ T cells.

**Conclusions:**

Results of the OPERA study demonstrate positive pharmacology and dose-dependent changes in pharmacodynamic biomarker measurements in blood, including changes in cellular composition of lymphocytes and corresponding CCR9 gene expression changes.

## 1. Introduction

Inflammatory bowel disease [IBD] is an extremely heterogeneous chronic inflammatory condition of the gastrointestinal tract, composed of two major disorders: ulcerative colitis [UC] and Crohn’s disease [CD]. Currently, therapeutic drug monitoring is being used to optimise biologic therapy. However, inter-patient heterogeneity in downstream pharmacology has limited the development of concrete thresholds for clinical management. Taken together, the definition of robust biomarkers of downstream pharmacology could be of tremendous value in optimising drug dosing. Whereas intestinal biomarkers would be most informative of relevant drug pharmacology, blood biomarkers are more tractable for routine clinical use. Unfortunately, questions remain about whether such blood biomarkers reflect local intestinal or broader systemic pharmacology, for the majority of drugs. However, antibodies blocking intestinal leukocyte trafficking may present a unique opportunity to define relevant pharmacodynamic [PD] biomarkers directly from the peripheral blood compartment, as these drugs prevent the influx of pathogenic immune cells into the intestine, trapping them in the systemic circulation.

PF-00547659 is a monoclonal antibody in development for the treatment of UC and CD. It is designed to prevent influx of pathogenic immune cells into the intestine by blocking the interaction of mucosal addressin cell adhesion molecule-1 [MAdCAM-1] on endothelial cells with β7 heterodimers on immune cells,^1^ thereby inhibiting extravasation of β7+ cells from the circulation to the gut. Blocking the ligand-receptor interaction with an anti-MAdCAM-1 agent is expected to increase frequency of β7+ cells in the circulation. In a recent Phase 2 randomised, double-blind, controlled study, PF-00547659 was found to be superior to placebo in inducing clinical remission/response and mucosal healing in patients with moderate to severe UC who had failed to respond to or were intolerant of at least one previous treatment [TURANDOT study; ClinicalTrials.gov, NCT01620255].^2^ Although other treatments have demonstrated clinical efficacy in both UC and CD (eg anti-tumour necrosis factor [TNF]α antagonists and anti-integrins [eg vedolizumab]), PF-00547659 did not demonstrate a statistically significant clinical treatment effect in the prospective efficacy endpoints in a Phase 2 randomised, double-blind, controlled study in patients with moderate to severe active CD [OPERA study; Clinicaltrials.gov, NCT01276509].^3^ In the OPERA study, biospecimens were collected from patients with CD to assess treatment effects of PF-00547659 on a number of PD biomarkers, including peripheral blood β7+ T cell populations, whole blood gene expression, and soluble MAdCAM [sMAdCAM] concentration in serum. Here, we present the results of these analyses that define surrogate pharmacology biomarkers in patients with CD after PF-00547659 treatment.

## 2. Methods

### 2.1. Study design

This Phase 2 clinical trial was a 12-week, randomised, double-blind, placebo-controlled, parallel-group study conducted to evaluate the efficacy and safety of the fully human immunoglobulin [Ig] G2қ anti-human MAdCAM-1 monoclonal antibody PF-00547659 in CD. Trial methodology has been described in detail in the previous publication of primary safety and efficacy findings.^3^ In brief, eligible adults were aged 18 to 75 years and had active moderate-to-severe CD (Crohn’s Disease Activity Index [CDAI] 220–450), a history of failure or intolerance with anti-TNF and/or immunosuppressive agents, high-sensitivity C-reactive protein [hsCRP] levels > 3.0 mg/l, and ulcers on colonoscopy [performed at baseline]. Patients were randomised in a 1:1:1:1 ratio and double-blind fashion to receive matching subcutaneous injections of placebo or 22.5 mg, 75 mg, or 225 mg of PF-00547659 [Pfizer, New York, NY] at baseline and Weeks 4 and 8, and were followed through Week 12. Azathioprine, 6-mercaptopurine, and methotrexate were continued at stable doses from screening through Week 8; dosages of these immunosuppressive agents were tapered by approximately 25% per week, beginning at Week 8, and were discontinued by Week 12.

The primary efficacy endpoint of the OPERA study was CDAI-70 clinical response [ie a decrease from baseline in CDAI ≥ 70 points] at Week 8 or Week 12. Secondary efficacy outcomes measured at all visits included CDAI-70 response, CDAI-100 response [ie a decrease from baseline in CDAI ≥ 100 points], CDAI remission [ie CDAI < 150], and mean change from baseline in total CDAI. Biospecimens used for these analyses were collected at various time points and at the Week 12 follow-up visit.

### 2.2. Determination of sMAdCAM in human serum

sMAdCAM was analysed at Weeks 2, 10, and 12. It was measured in serum using a highly specific immunoaffinity liquid chromatography tandem mass spectrometry [IA-LC-MS/MS] assay with high specificity and sensitivity [detailed methodology described in Supplementary Appendices, Appendix 1, available as Supplementary data at *ECCO-JCC* online].^4^^,^^5^

### 2.3. Flow cytometric assays

Fluorescence-activated cell sorting [FACS] was used to measure molecules of equivalent soluble fluorochrome [MESF: a unit that accesses the expression of β7 per cell] and percentage of β7+ and β7 negative central memory T cells, β7+ effector memory T cells, and β7+ naïve T cells [Table 1; Supplementary Table 1, available as Supplementary data at *ECCO-JCC* online; and Supplementary Appendices, Appendix 1] from blood samples drawn into sodium heparin BD Vacutainers^®^ [BD Life Sciences, Franklin Lakes, NJ] at baseline, Week 8, and Week 12. The gating strategy for the FACS β7-integrin assay using blood samples from a healthy volunteer and an OPERA study patient with CD is shown in Supplementary Figure 1A and 1B, available as Supplementary data at *ECCO-JCC* online.

**Table 1.** Population description of cells analysed by FACS.

FACS population	Description
β7 negativeCD45RO+CD27+CD3+CD4+	β7 integrin negative central memory T cells
β7+CD45RO+CD27+CD3+CD4+	β7 integrin positive central memory T cells
β7+CD45RO+CD27-CD3+CD4+	β7 integrin positive effector memory T cells
β7+CD45RO-CD27+CD3+CD4+	β7 integrin positive naïve T cells

For details of phenotype, see Supplementary Table 1, available as Supplementary data at *ECCO-JCC* online.

FACS, fluorescence-activated cell sorting.

### 2.4. Association between β7+ T cell response and PF-00547659 plasma levels

Blood samples for the assessment of PF-00547659 concentrations were collected and analysed at specified time points for the duration of the study, using a validated assay. To better understand PF-00547659 concentration-effect relationship in the prevention of β7+ expressing cells entering the gut mucosa, trough plasma concentrations of PF-00547659 at Week 12 across all dose groups were divided into four quartiles: Q1, median 271 ng/ml [range: 0–1132.5 ng/ml]; Q2, median 2140 ng/ml [range: 1132.5–5205.0 ng/ml]; Q3, median: 7070 ng/ml [range: 5205.0–12825.0 ng/ml]; and Q4, median: 18800 ng/ml [range: 12825.0–40800.0 ng/ml].

### 2.5. Gene expression profiling analysis

Peripheral venous blood samples were collected from patients at baseline and Week 12 for gene expression profiling analysis [detailed methodology described in Supplementary Appendices, Appendix 2, available as Supplementary data at *ECCO-JCC* online].^4^^,^^6–11^

### 2.6. Statistical analysis

The geometric means for flow cytometry parameters, sMAdCAM, faecal calprotectin, and hsCRP were summarised descriptively. The geometric means and percentage change from baseline in geometric means were calculated and plotted for each treatment group over time with 90% confidence interval [CI]. This Phase 2 study was designed using a type 1 error of 5% [one-sided]; a 90% CI was calculated to provide a CI that would be aligned with the type 1 error used in the design stage. Inferential statistics were also produced where the flow cytometry parameters were log transformed and analysed using a linear mixed model that included change from baseline as response, treatment, status of anti-TNF experience, concomitant immunosuppressant therapy, baseline [log transformed] visit, and treatment-by-visit interaction as fixed effects, and patients as random effect. Logarithmic [two-base] counts per million mapped reads were used as a measure of gene expression for statistical analyses. For each biomarker including sMAdCAM, β7+ cells, and gene expression, change scores between baseline and Week 12 were calculated and analysed based on the different endpoint assessed [Supplementary Appendices, Appendix 3, available as Supplementary data at *ECCO-JCC* online].^12^

## 3. Results

Of 494 screened patients, 265 were eligible for study entry and 262 were randomised and treated [placebo, *n* = 63; PF-00547659, 22.5 mg, *n* = 66; 75 mg, n = 65; and 225 mg, *n* = 68]. Baseline characteristics and demographics of the patient population are shown in Table 2.^3^

**Table 2.** Baseline demographic and disease characteristics.^3
^

	Placebo	PF-00547659 22.5 mg	PF-00547659 75 mg	PF-00547659 225 mg
*N*	63	66	65	68
Age, years, mean [SD]	34.4 [11.1]	37.3 [13.0]	34.4 [10.7]	35.9 [11.0]
Sex, female, *n* [%]	30 [47.6]	48 [72.7]	35 [53.8]	43 [63.2]
Race, *n* [%]				
White	54 [85.7]	53 [80.3]	53 [81.5]	60 [88.2]
Black	1 [1.6]	2 [3.0]	2 [3.1]	2 [2.9]
Asian	5 [7.9]	8 [12.1]	8 [12.3]	6 [8.8]
Other	3 [4.8]	3 [4.5]	2 [3.1]	0
Weight, kg, mean [SD]	70.1 [19.4]	71.9 [17.5]	69.5 [21.5]	69.6 [20.9]
Disease duration, years, mean	11.5	12.7	11.4	12.0
hsCRP, mg/l, median [range]	18.9 [2.3–240.9]	21.1 [1.3–178.0]	14.7 [0.3–180.1]	17.2 [2.4–117.3]
CDAI, mean [SD]	313.1 [61.4]	307.4 [71.1]	324.4 [63.1]	316.4 [64.6]
Anti-TNF therapy experience, *n* [%]				
Relapsed after ≥ 1 anti-TNFα	34 [54.0]	34 [51.5]	37 [56.9]	39 [57.4]
No response to ≥ 1 anti-TNFα	12 [19.0]	13 [19.7]	11 [16.9]	11 [16.2]
Intolerant to ≥ 1 anti-TNFα	12 [19.0]	13 [19.7]	12 [18.5]	13 [19.1]
Failure/intolerance to any immunosuppressant	5 [7.9]	6 [9.1]	5 [7.7]	5 [7.4]
Current use of corticosteroids, *n* [%]	29 [46.0]	31 [47.0]	36 [55.4]	35 [51.5]
Use of immunosuppressant therapy at study entry, *n* [%]				
Azathioprine	13 [20.6]	11 [16.7]	15 [23.1]	15 [22.1]
6-mercaptopurine	2 [3.2]	6 [9.1]	6 [9.2]	4 [5.9]
Methotrexate	6 [9.5]	10 [15.2]	7 [10.8]	7 [10.3]
No immunosuppressives	42 [66.7]	39 [59.1]	37 [56.9]	42 [61.8]
Central memory CD4+ T cells^a^ MESF, median [IQR]	638.5 [117–2647]	670.0 [45–1669]	877.5 [260–2402]	660.5 [193–2252]

CDAI, Crohn’s Disease Activity Index; hsCRP, high-sensitivity C-reactive protein; IQR, interquartile range; MESF, molecules of equivalent soluble fluorochrome; SD, standard deviation; TNF, tumour necrosis factor.

^a^Data not available from all patients, therefore *n* values are smaller than for total population [*n* = 47, *n* = 47, *n* = 43, *n* = 53 for placebo, and PF-00547659 22.5 mg, 75 mg, and 225 mg, respectively].

### 3.1. Treatment-related changes in sMAdCAM

**Figure 1.**
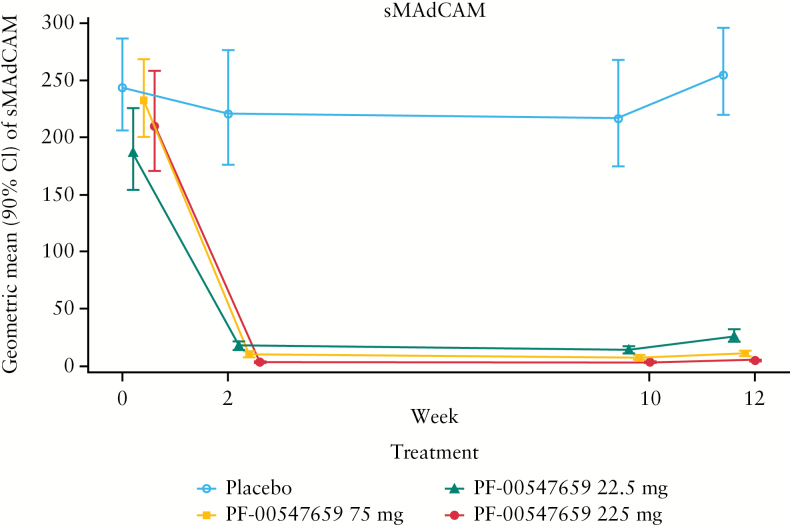
The time course of serum soluble MAdCAM levels [geometric mean 90% CI, ng/ml] in patients following treatment with placebo or PF-00547659. Different symbols represent different dose groups: ○ placebo, ▲ PF-00547659 22.5 mg, ■ PF-00547659 75 mg, and ● PF-00547659 225 mg. CI, confidence interval; sMAdCAM, soluble mucosal addressin cell adhesion molecule.

Because intestinal tissue biopsies are difficult to obtain, we developed an sMAdCAM assay for the blood as a surrogate marker for target engagement at the site of action in the intestine. Decreases in sMAdCAM were observed with active treatment starting at Week 2. The geometric mean [90% CI] percent changes from baseline in sMAdCAM data are shown in Figure 1. Whereas the percent changes in the placebo group increased slightly [5.8%] at Week 12, all of the active treatment groups showed significant decreases in sMAdCAM, ranging from –87.1% to –97.7%. The decreases in geometric mean percent changes from baseline in sMAdCAM at Week 12 for the PF-00547659 22.5-mg, 75-mg, and 225-mg doses were –87.1% [–90.4%, –82.6%], –95.4% [–96.4%, –94.2%], and –97.7% [–98.2%, –97.1%], respectively.

### 3.2. Changes in central memory, effector memory, and naïve T cells

**Figure 2.**
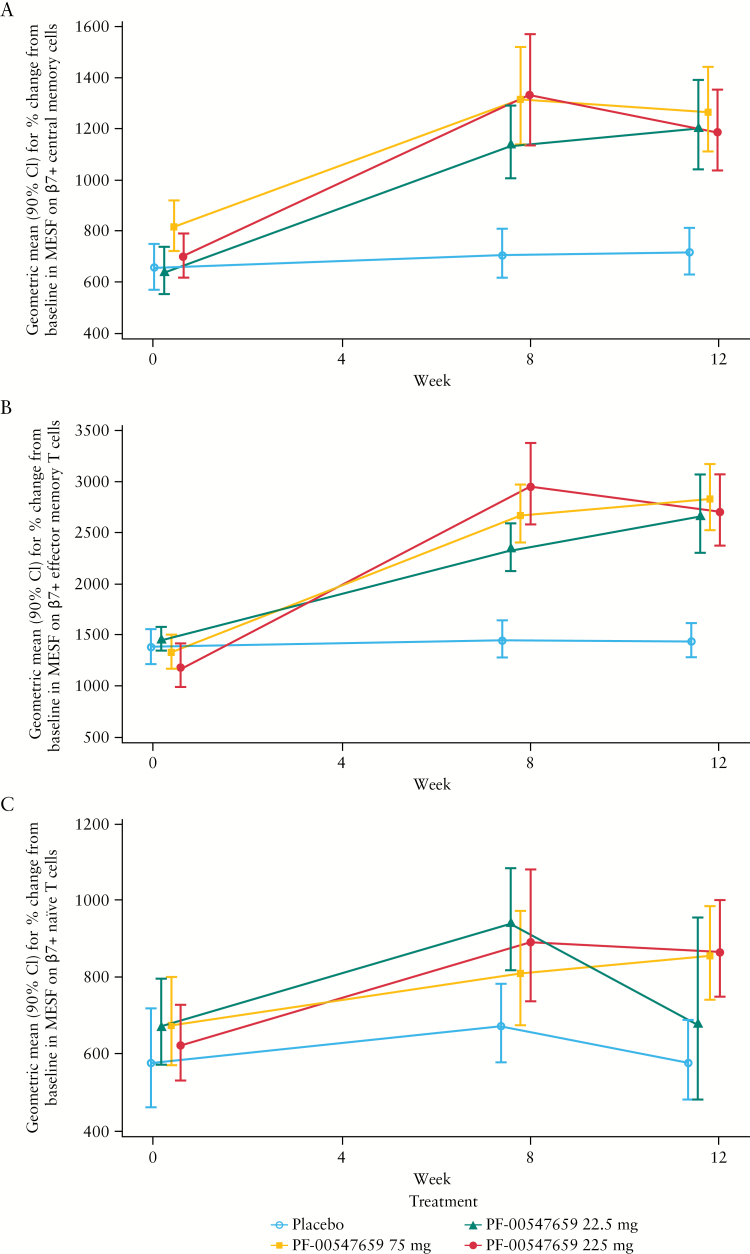
Effect of PF-00547659 on [A] β7+ central memory T cells; [B] β7+ effector memory T cells; and [C] β7+ naïve T cells, in CD4+ cell subsets of patients with CD. β7 expression was measured by a validated whole blood assay [see Supplementary Figure 1 for gating strategy]. The error bars are geometric mean estimates [90% CI] for percent change from baseline in MESF. CI, confidence interval; MESF, molecules of equivalent soluble fluorochrome.

The geometric mean [90% CI] estimates for percent change from baseline in MESF on β7+ central memory cells are shown in Figure 2A. The geometric mean for percent change from baseline to Week 12 for the placebo group increased to 4.1%. The drug-induced increases for MESF for the central memory cells at Week 12 ranged from 48.0% to 80.7%. The increases were statistically significant for both Week 8 and Week 12. The geometric mean [90% CI] estimates for percentage change from baseline in percent β7+ central memory cells are shown in Supplementary Figure 2A, available as Supplementary data at *ECCO-JCC* online. The geometric mean for percent change from baseline for percent β7+ central memory cells at Week 12 for the placebo group increased to 5%. The drug-induced increases for percent β7+ central memory cells at Week 12 ranged from 24.1% to 39.5%. The increases were statistically significant for both Week 8 and Week 12.

The geometric mean [90% CI] estimates for percent change from baseline in MESF on β7+ effector memory T cells are shown in Figure 2B. For effector memory T cells, whereas the mean in the placebo group increased slightly (8.0% [–6.4, 24.6]) at Week 12, all of the active groups showed statistically significant increases in MESF on β7+ effector memory T cells. The increases in geometric mean percent changes from baseline in MESF of effector memory T cells at Week 12 for the 22.5-mg, 75-mg, and 225-mg doses were 92.3% [67.9, 120.2], 83.8% [57.4, 114.6], and 138.2% [91.0, 197.0], respectively. The geometric mean [90% CI] estimates for percentage change from baseline in percent β7+ effector memory T cells are shown in Supplementary Figure 2B, available as Supplementary data at *ECCO-JCC* online. For effector memory T cells, all of the active treatment groups, except the 22.5-mg group, showed an increase in percent β7+ effector T cells at Week 8. At Week 12, the active treatment groups had increases ranging from 3.3% to 14.0%.

When analysed using the linear mixed model [as described in the statistical analysis section] at Week 8, the relative ratios of MESF on β7+ effector memory T cells for the 22.5-mg, 75-mg, and 225-mg PF-00547659 doses versus placebo were 1.554, 1.792, and 1.992, respectively [Supplementary Figure 3A, available as Supplementary data at *ECCO-JCC* online]. The relative ratios for MESF on β7+ effector memory T cell estimates were statistically significant for all doses at Week 8. At Week 12, the relative ratios to placebo for 22.5 mg, 75 mg, and 225 mg PF-00547659 were 1.822, 1.871, and 1.932, respectively [Supplementary Figure 3A]. The relative ratios of percent β7+ effector memory T cells to placebo are shown in Supplementary Figure 4A, available as Supplementary data at *ECCO-JCC* online.

The geometric mean [90% CI] estimates for percent change from baseline in MESF on β7+ naïve T cells are shown in Figure 2C. Whereas MESF in the placebo group increased slightly (7.7% [–18.1, 41.6]) at Week 12, all of the active treatment groups showed increases in MESF on β7+ naïve T cells, ranging from 12.8% to 49.8%. The increases in geometric mean for percent change from baseline in percent β7+ naïve T cells at Week 12 for the 22.5-mg, 75-mg, and 225-mg doses were 12.8% [–21.9%, 63.0%], 49.8% [15.7%, 93.9%], and 31.1% [10.2%, 56.1%], respectively, and are shown in Supplementary Figure 2C. Changes in the percent β7+ naïve T cells over time were highly variable across the treatment groups, and there were no apparent treatment effects. The placebo group remained stable over 12 weeks.

In addition, the MESF data were analysed using a linear mixed-effects model, shown in Supplementary Figure 3B. Fold changes in MESF were only statistically significant for the 75-mg and 225-mg doses at Week 12. The relative ratios of percent β7+ naïve T cells to placebo are also shown in Supplementary Figure 4B, available as Supplementary data at *ECCO-JCC* online.

**Figure 3.**
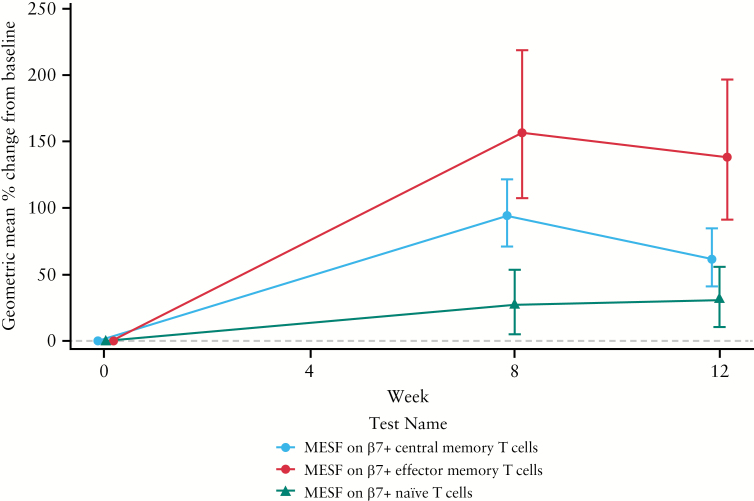
Effect of PF-00547659 on percent change from baseline of β7 expression in CD4+ cell subsets of patients with CD in the 225-mg group. Geometric mean % MESF change from baseline on β7+ in central memory, effector memory, and naïve T cells are calculated from data in Figure 2. CD, Crohn’s disease; FACS, fluorescence-activated cell sorting; MESF, molecules of equivalent soluble fluorochrome.

The MESFs of central memory, effector memory, and naïve T cells for the PF-00547659 225-mg group are compared in Figure 3. As shown, the drug had a greater effect on MESF values of β7+ cells in central and effector memory subsets than on naïve T cells.

Lastly, longitudinal monitoring of β7 integrin negative central memory T cells [β7 negativeCD45RO+CD27+CD3+CD4+] showed no change in this population [data not shown].

### 3.3. Associations among serum hsCRP levels, serum PF-00547659 levels, and clinical response and remission

At baseline, hsCRP levels were highly variable among individual patients, ranging from 0.33 to 240.9 mg/l. Median hsCRP levels at baseline were 18.9, 21.1, 14.7, and 17.2 mg/l in the placebo, 22.5-mg, 75-mg, and 225-mg dose groups, respectively; hsCRP levels at the end of treatment [Week 12] were 19.9, 11.8, 9.9, and 15.6 mg/l in these groups, respectively.

As previously reported,^3^ findings from an analysis of the systemic exposure-response relationship for hsCRP showed no correlation between serum hsCRP and PF-00547659 levels at Week 12. In addition, serum exposure to PF-0547659 was not related to clinical response [ie CDAI-70 or CDAI-100 response^3^] or remission [CDAI < 150] [Supplementary Figure 5, available as Supplementary data at *ECCO-JCC* online]. Only minimal differences were seen in median changes in hsCRP levels from baseline between patients who did and did not achieve clinical response or remission at Week 12 [data not shown].

### 3.4. Associations among faecal calprotectin levels, serum PF-00547659 levels, and clinical response and remission

Faecal calprotectin levels were highly variable among individuals at baseline, ranging from 22.8 to 31,588 μg/g; median levels at baseline were similar among treatment groups, averaging 1797, 1705, 1389, and 1346 μg/g in the placebo, 22.5-mg, 75-mg, and 225-mg PF-00547659 dose groups, respectively. After 12 weeks, faecal calprotectin levels were 1678, 987, 1066, and 1769 μg/g in the respective treatment groups.

No relationship was observed between systemic exposure to PF-00547659 and the degree of intestinal inflammation as measured by faecal calprotectin levels at Week 12.^3^ In addition, no correlation was seen between changes in faecal calprotectin levels from baseline to Week 12 and the proportions of patients achieving clinical response or remission, as median faecal calprotectin levels were only minimally different between patients who did and did not achieve these outcomes [data not shown].

### 3.5. Association between β7+ T cell response and PF-00547659 plasma levels

The percent changes from baseline to Week 12 in MESF of total β7+ and β7 negative T cells, β7+ and β7 negative central memory T cells, β7+ effector memory T cells, and β7+ naïve T cells across placebo and the three PF-00547659 dose groups by quartile are shown in Supplementary Figure 6, available as Supplementary data at *ECCO-JCC* online. The median percent change in β7+ T cells in blood was consistently higher in each of the quartiles of pharmacokinetic [PK] levels of PF-00547659 compared with the placebo group for the number of total β7+ [Supplementary Figure 6A], central memory [Supplementary Figure 6C], effector memory [Supplementary Figure 6E], and naïve total cell population [Supplementary Figure 6F], with the mean percentage of β7+ T cells increasing from the first to the third quartile. For the central and effector memory T cells, the increase in β7+ cells in the blood was similar across all quartiles and the maximum treatment effect was reached at a median concentration of 271 ng/ml. The median percentage of change in cells that did not express the β7 receptor [β7 negative T cells] on their surface [total T cells or central memory T cells] in blood did not differ between the placebo and PF-00547659 dose groups [Supplementary Figure 6B, Supplementary Figure 6D].

### 3.6. Transcriptional changes in response to treatment with PF-00547659

**Figure 4.**
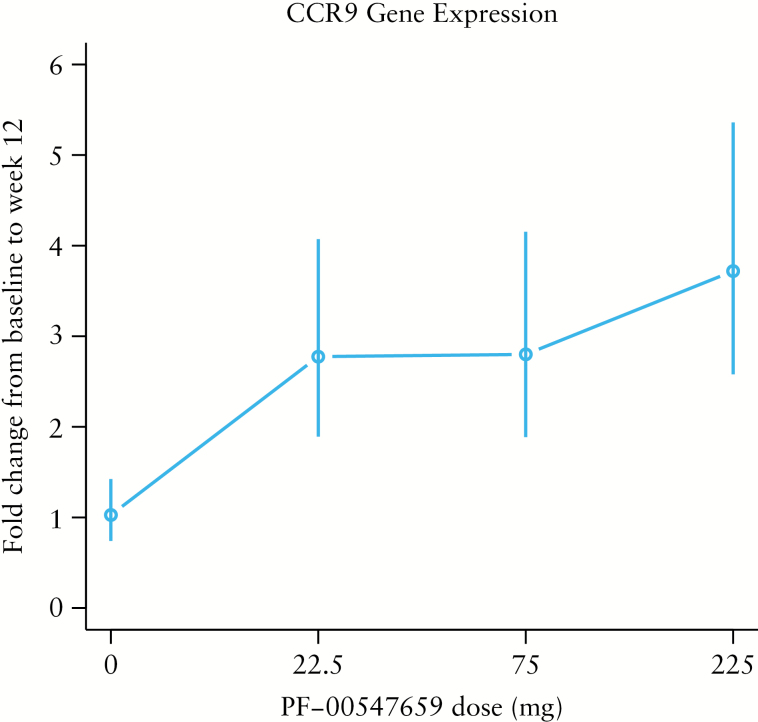
Fold changes in CCR9 gene expression [measured by counts per million] from baseline to Week 12 by treatment group. Each vertical line represents the 90% confidence interval for each fold change.

Expression of the CCR9 gene was increased from baseline to Week 12 in the treatment groups. This increase was statistically significant, with a *p*-value of 1.09e-06 and a false discovery rate [FDR] < 0.1 [Figure 4]. Fold changes in the placebo and the 22.5-mg, 75-mg, and 225-mg dose groups were 1.03, 2.78, 2.80, and 3.72, respectively. No genes were found to be significantly associated with treatment response [CDAI 70] or β7 surface protein expression measured by FACS with an FDR < 0.1. CCR9 expression and sMAdCAM levels were negatively correlated [Spearman correlation, rho = –0.58] [Supplementary Figure 7, available as Supplementary data at *ECCO-JCC* online].

After not observing significant individual gene expression changes with an FDR < 0.1, the top 2000 genes associated with treatment response in any dose group with *p*-values < 0.1 [as reported in Supplementary Table 2, available as Supplementary data at *ECCO-JCC* online] were used to gain insights into the mechanism of action of PF-00547659 on a broader pathway level. Many of the most significant canonical pathways and process networks enriched in these 2000 genes were centred around T cell-related processes such as TCR signalling, T helper cells, T cell co-stimulation, leukocyte extravasation signalling, cell adhesion and leukocyte chemotaxis, PI3K, and inositol phosphate metabolism, all suggesting modulation of T cell pathways by PF-00547659.

**Figure 5.**
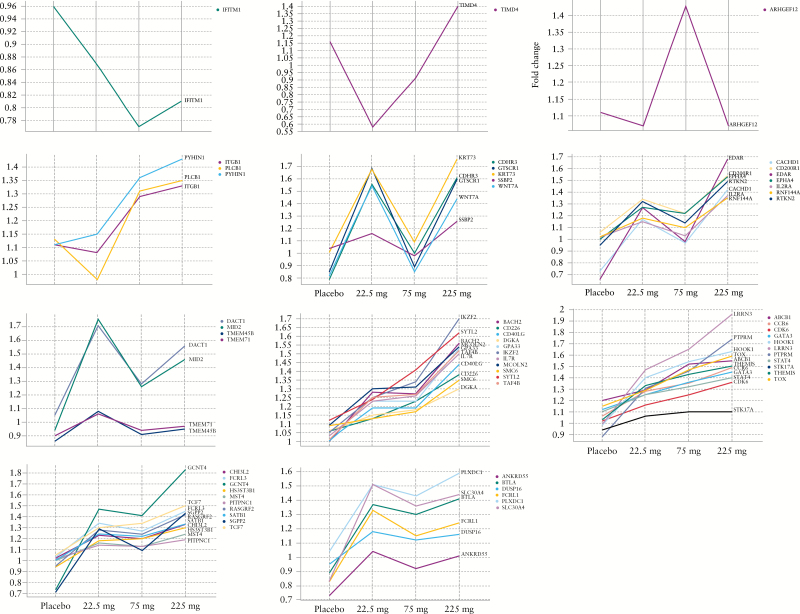
Expression profile of TReg-cell–related genes across treatment arms, ie in placebo, and PF-00547659 22.5-mg, 75-mg, and 225-mg groups, at Week 12. TReg, T regulatory cells.

Gene expression associated with T effector [Teff] cells, T central memory [Tcm] cells, and T regulatory [TReg] cells received particular attention, as these T cell subsets are believed to be involved in CD and they express α4β7. Genes involved in trans-endothelial migration and Teff and Tcm cells were also enriched in the 2000 genes. One of the most significant gene signatures identified in the set of 2000 genes included the FOXP3 target genes derived from MSigDb.^13^ The enrichment for TReg genes was confirmed using additional human FOXP3 target gene sets and TReg gene signatures,^8–10^ thereby suggesting a modulation of TRegs by PF-00547659. Furthermore, FOXP3 was predicted to be an upstream modulator of the observed gene-expression changes [*p*-value 7.94E-4]. The majority of the 60 TReg genes represented in the 2000 genes, such as LRRN3 [previously shown to be up-regulated in β7+ versus β7 negative CD4+ CD45RA– T-helper cells^14^] share the trends observed for the treatment effects on β7+ central memory T cells in patients’ blood, with the exception of ARHGEF12, PLCB1, and TIMD4, and a few others [Figure 5], suggesting a similar effect of PF-00547659 in increasing circulating TReg cell levels. All enrichment analysis results are shown in Supplementary Table 2.

**Figure 6.**
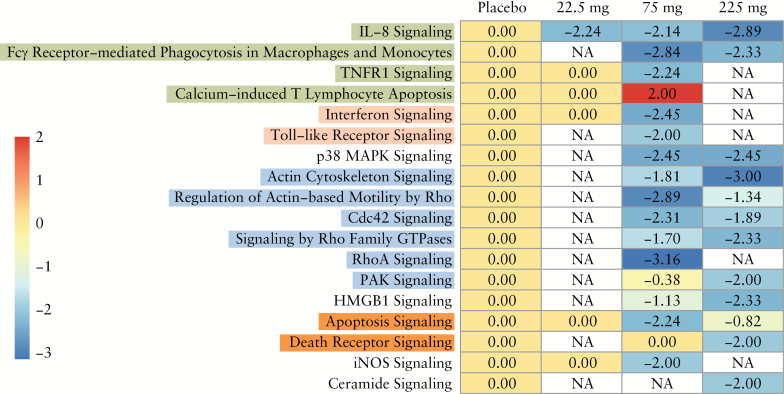
Canonical pathways enriched in each treatment group with a z-score ≥ 2 or ≤ –2 and –log [*p*-value] > 1.3. Enriched pathways with z-scores of ≥ 2 were activated by differentially expressed genes, whereas those with z-scores ≤ –2 indicated inhibition of the enriched pathways. All enriched pathways met the statistical significance cut-off of *p* < 0.05.

Interleukin [IL]-8 signalling was the only statistically significant pathway in all three active treatment groups and could have been predicted to be down-regulated with PF-00547659 treatment. No dose-dependent modulation of pathways other than IL-8 was noted based on the canonical pathways enriched in any of the treatment arms with a Z-score of ≥ 2 or ≤ –2 and –log [*p*-value] > 1.3 [Figure 6].

## 4. Discussion

β7+ T cells and gene expression of CCR9 were demonstrated as surrogate biomarkers of PD activity in this Phase 2 study in patients with moderate to severe CD, using blood biomarkers. Within the blood, a number of different cell types have been found to express the β7+ integrin receptor. In this study, we measured the three most abundant cell types, ie β7-expressing central memory T cells, effector memory T cells, and naïve T cells. Our analyses demonstrate an increase in all three β7+ T cell types [MESF levels] with PF-00547659 treatment, even at the lowest dose [22.5 mg]. The proposed mechanism of action for PF-00547659 is inhibition of immune cell trafficking into the intestine by blockade of the critical interaction between β7 and MAdCAM-1. Continued blockade of this interaction in the intestine should result in increased numbers of β7+ cells in the peripheral blood. Therefore, our finding of increased peripheral β7+ T cells confirms the proposed mechanism of action and supports a pharmacological effect of PF-00547659 treatment.

To confirm that the increase in number of β7-expressing T cells after PF-00547659 treatment was related to the mechanism of action of the drug and not only to increases in the overall population of cells in blood, FACS analysis was performed on the same cell population without expression of the β7 receptor, ie the β7 negative cell population. The number of β7 negative T cells was not changed over time in either the placebo or the PF-00547659 treatment group. This important finding demonstrates that the increase in number of β7-expressing T cells is related to the mechanism of action of the drug. When assessing the various cell types that express the β7 receptor, β7+ central memory and effector T cells were the most sensitive subset of T cells demonstrating a treatment effect in all PF-00547659-treated patients at Week 12 when compared with baseline, as both frequency [%] and MESF were increased by treatment. The mechanism of increased MESF in circulating β7+ cells is unclear, but one potential explanation is the mobilisation of high β7 expressors from the gastrointestinal tract to the circulation after PF-00547659 treatment. Interestingly, PF-00547659 seems to have a greater effect on circulating β7-expressing cells in normal monkeys^1^ than in patients with CD [Figure 2]. It is unclear whether such difference is associated with species or diseases.

Gene expression profiling analyses revealed significant up-regulation of CCR9 from baseline to Week 12 in the circulation in all treatment groups, but no significant changes in other genes. Both CCR9 and β7 are intestinal-homing receptors expressed on lymphocytes, which target the intestine through CCR9-CCL25 and β7-MAdCAM-1 interaction, respectively. CCL25 and MAdCAM-1 are constitutively expressed on intestinal capillary venules and can recruit CCR9+ and β7+ lymphocytes from the circulation.^15^ Whereas whole blood transcriptomics measured CCR9 expression on a wide variety of different gut-homing leukocytes, such as T cells and B cells, our FACS analysis focused only on gut-homing T cells. It is therefore not surprising that the correlation between blood-based CCR9 gene expression and β7+ T cell numbers measured by FACS is fairly low [Spearman correlation rho = 0.37; *p* = 0.0032]. To our knowledge, the increase in both β7+ CD4 T cells and CCR9 gene expression has not been reported previously. As whole blood CCR9 transcript measurements are easy to perform and more robust than β7 measurements by FACS, this has particular relevance for PD biomarker assessment in multicentre clinical trials, as well as eventual clinical usage.

Gene set enrichment analysis shows a significant enrichment of many T cell-related pathways and processes modulated by PF-00547659, including TRegs. Interestingly, most of the TReg genes show an expression profile similar to α4β7+ central memory T cells, with the exception of a few genes. TRegs, similarly to other CD4+ T cell populations, also express β7 markers and it is therefore not surprising that, overall, they behave similarly in their response to PF-00547659 therapy. Based on this, we hypothesise that PF-00547659 has a similar effect on TReg cells consistent with the observed effect on β7+ central memory T cells. This finding could have significant implications in defining optimal therapeutic doses.

We did not observe any commonalities for pathway modulation across the three dose groups with the exception of the IL-8 signalling pathway. Key components of the IL-8 pathway include CXCR1, the receptor for IL-8, and kinases such as PAK1, LIMK1, and LIMK2. IL-8 signalling is involved in chemotaxis of multiple immune cell types, including T cells, neutrophils, and macrophages, resulting in inflammation, angiogenesis, and other biological processes. Interestingly, IL-8 has also been shown to promote integrin β7-mediated adhesion to VCAM1 and suppress adhesion to MAdCAM-1, thereby playing a role in T cell homing to different tissues.^6^ Genes downstream of CXCR1 that are down-regulated by PF-00547659 treatment include proximal regulators of G-protein signalling [GNAI2, GNB2], Rho, Rac, MAP Kinase signalling, LIM kinase signalling NF-kB, ICAM1, and MMP9. A predicted reduction in IL-8 signalling based on the genes involved in this pathway suggests a reduction in T cell chemotaxis by PF-00547659.

As predicted by PK modelling, sMAdCAM levels markedly decreased by approximately 90% following all doses of PF-00547659, and the decreases were statistically significant. The sMAdCAM assay detects the fraction of sMAdCAM that is not bound to PF-00547659. Therefore, the observed dose-dependent reduction of sMAdCAM is predominantly a consequence of engagement of PF-00547659 with sMAdCAM in blood. In addition, binding of PF-00547659 to membrane MAdCAM-1 may prevent shedding to yield sMAdCAM. Thus, we believe that the reduction in sMAdCAM may serve as a demonstration of the pharmacological effect of PF-00547659.

The lack of clinical efficacy observed with PF-00547659 in the OPERA study has led to the suggestion that MAdCAM-1 blockade may only be effective in superficial colonic disease, ie UC. However, recent experience with vedolizumab, a humanised monoclonal antibody that targets the α4β7 integrin heterodimer and blocks β7 integrin-MAdCAM-1 interaction, suggests otherwise.^7^ The lack of clinical efficacy findings in OPERA may be explained by the study design, particularly by reliance on subjective measures [ie CDAI] rather than on more objective endoscopic measures to assess therapeutic efficacy, by the possible need for longer exposure time to see a response in CD, or by nuances in the therapeutic mechanism of action of the biologic. The bell-shaped dose-response curve, observed with PF-00547659 doses ranging from 7.5 mg to 225 mg in the Phase 2 clinical investigation in patients with UC,^16^ may indicate that excessive inhibition of the binding of β7 integrin to human MAdCAM-1 has an unintentionally negative impact on clinical response. Moreover, the plateau in sMAdCAM levels, changes in β7 expressing T cells, and increases in CCR9 gene expression in our study of patients with CD, suggest that the PF-00547659 doses tested could possibly have been too high, potentially leading to unwanted reduction of TReg activity in the inflamed colonic mucosa. Given these questions, further research into the clinical efficacy of this agent in CD may be warranted.

In conclusion, results of the OPERA study demonstrate PD activity based on changes in cellular composition of lymphocytes and CCR9 gene expression, which may have future utility for optimising therapy in the context of a broader therapeutic drug monitoring-based approach. To our knowledge, this is the first time the combination of comprehensive datasets for circulating lymphocytes and gene expression have been generated and analysed within an IBD clinical trial using an anti-MAdCAM-1 antibody. Our results showed that subtyping of lymphocytes in the circulation and measurement of CCR9 gene expression may be suitable surrogates of disease-relevant PD biomarkers, which are easier to conduct than intestinal tissue biopsy. These surrogates can be used to define relevant PD biomarkers directly from the more tractable peripheral blood compartment, as anti-MAdCAM-1 antibody prevents the influx of pathogenic immune cells into the intestine, trapping them in the systemic circulation. Additionally, based on our experience from this clinical study, we believe mRNA analysis is easier to implement than FACS. In the future, for classes of drugs that have a gut-cell trafficking mechanism, CCR9 gene expression could be considered as a better alternative PD biomarker than FACS analysis of circulating lymphocytes.

Experience with vedolizumab suggests the lack of clinical efficacy observed with PF-00547659 may not simply be a result of the differing disease biology in CD. Furthermore, additional clinical experience with PF-00547659 in UC suggests that an appropriate balance between competing pro- and anti-inflammatory mechanisms may be required for anti-MAdCAM-1 drugs to achieve optimal clinical efficacy. Additional clinical studies may be needed, with more objective endoscopic endpoints, to fully define the efficacy of anti-MAdCAM-1 therapy in CD.

## Funding

The OPERA study was funded by Pfizer.

## Conflict of Interest

MH-Z, AB, WZ, KP, DvS, BZ, SWM, SN, PR, LX, HN, MFO, MV, and KH are employees of Pfizer. JBC, AA, KG, RC, and FC were employees of Pfizer during the OPERA study.

## Author Contributions

KH, AB, JBC, WZ, KP, DvS, BZ, SWM, SN, PR, LX, HN, MFO, KG, RC, MV, and MH-Z contributed to the design of the analyses, interpretation of the data, and drafting of the manuscript. As guarantor, MH-Z was responsible for the overall content of the manuscript in addition to design and strategy of the biomarker plan, analysis and interpretation of the data, and drafting the manuscript. All authors reviewed and revised the manuscript for important intellectual content and approved the final version of the manuscript before its submission.

## Supplementary Data

Supplementary data are available at *ECCO-JCC* online.

## Supplementary Material

Supplementary Figure S1

Supplementary Figure S2

Supplementary Figure S3

Supplementary Figure S4

Supplementary Figure S5

Supplementary Figure S6

Supplementary Figure S7

Supplementary Appendices

## References

[1] Pullen N, Molloy E, Carter D Pharmacological characterization of PF-00547659, an anti-human MAdCAM monoclonal antibody. Br J Pharmacol 2009;157:281–93. 19366349 10.1111/j.1476-5381.2009.00137.x PMC2697799

[2] Vermeire S, Sandborn WJ, Danese S Anti-MAdCAM antibody [PF-00547659] for ulcerative colitis [TURANDOT]: a phase 2, randomised, double-blind, placebo-controlled trial. Lancet 2017, May 17. doi: 10.1016/S0140-6736[17]30930–3. [Epub ahead of print.] 10.1016/S0140-6736(17)30930-3 28527704

[3] Sandborn WJ, Lee S, Tarabar D A Phase 2 evaluation of anti-MAdCAM antibody PF-00547659 in the treatment of Crohn’s disease: Report of the OPERA study. Gut 2017 [in press]. 10.1136/gutjnl-2016-313457 PMC6145284 28982740

[4] Palandra J, Finelli A, Zhu M, Masferrer J, Neubert H Highly specific and sensitive measurements of human and monkey interleukin 21 using sequential protein and tryptic peptide immunoaffinity LC-MS/MS. Anal Chem 2013;85:5522–9. 23638938 10.1021/ac4006765

[5] Neubert H, Muirhead D, Kabir M, Grace C, Cleton A, Arends R Sequential protein and peptide immunoaffinity capture for mass spectrometry-based quantification of total human β-nerve growth factor. Anal Chem 2013;85:1719–26. 23249404 10.1021/ac303031q

[6] Sun H, Liu J, Zheng Y, Pan Y, Zhang K, Chen J Distinct chemokine signaling regulates integrin ligand specificity to dictate tissue-specific lymphocyte homing. Dev Cell 2014;30:61–70. 24954024 10.1016/j.devcel.2014.05.002

[7] Singh H, Grewal N, Arora E, Kumar H, Kakkar AK Vedolizumab: A novel anti-integrin drug for treatment of inflammatory bowel disease. J Nat Sci Biol Med 2016;7:4–9. 27003961 10.4103/0976-9668.175016 PMC4780165

[8] Dobin A, Davis CA, Schlesinger F STAR: ultrafast universal RNA-seq aligner. Bioinformatics 2013;29:15–21. 23104886 10.1093/bioinformatics/bts635 PMC3530905

[9] Quinlan AR, Hall IM BEDTools: a flexible suite of utilities for comparing genomic features. Bioinformatics 2010;26:841–2. 20110278 10.1093/bioinformatics/btq033 PMC2832824

[10] Robinson MD, McCarthy DJ, Smyth GK edgeR: a bioconductor package for differential expression analysis of digital gene expression data. Bioinformatics 2010;26:139–40. 19910308 10.1093/bioinformatics/btp616 PMC2796818

[11] Chindelevitch L, Ziemek D, Enayetallah A Causal reasoning on biological networks: interpreting transcriptional changes. Bioinformatics 2012;28:1114–21. 22355083 10.1093/bioinformatics/bts090

[12] Dalmasso C *LBE: Estimation of the False Discovery Rate*. R package version 1.26.0. 2007 http://citeseerx.ist.psu.edu/viewdoc/download?doi=10.1.1.304.829&rep=rep1&type=pdf Accessed December 9, 2015.

[13] Subramanian A, Tamayo P, Mootha VK Gene set enrichment analysis: a knowledge-based approach for interpreting genome-wide expression profiles. Proc Natl Acad Sci U S A 2005;102:15545–50. 16199517 10.1073/pnas.0506580102 PMC1239896

[14] Rodriguez MW, Paquet AC, Yang YH, Erle DJ Differential gene expression by integrin beta 7+ and beta 7- memory T helper cells. BMC Immunol 2004;5:13. 15236665 10.1186/1471-2172-5-13 PMC476736

[15] Gorfu G, Rivera-Nieves J, Ley K Role of beta7 integrins in intestinal lymphocyte homing and retention. Curr Mol Med 2009;9:836–50. 19860663 10.2174/156652409789105525 PMC2770881

[16] Vermeire S, Sandborn WJ, Danese S Anti-MAdCAM antibody [PF-00547659] for ulcerative colitis: the TURANDOT study. Lancet 2017;390:135–44. 28527704 10.1016/S0140-6736(17)30930-3

