# Supplementary figures and images for: Anti-MAdCAM Antibody Increases ß7+ T Cells and CCR9 Gene Expression in the Peripheral Blood of Patients With Crohn’s Disease
Source: J Crohns Colitis. 2017 Sep 7;12(1):77–86. doi: 10.1093/ecco-jcc/jjx121 (PMC5881777; doi:10.1093/ecco-jcc/jjx121)

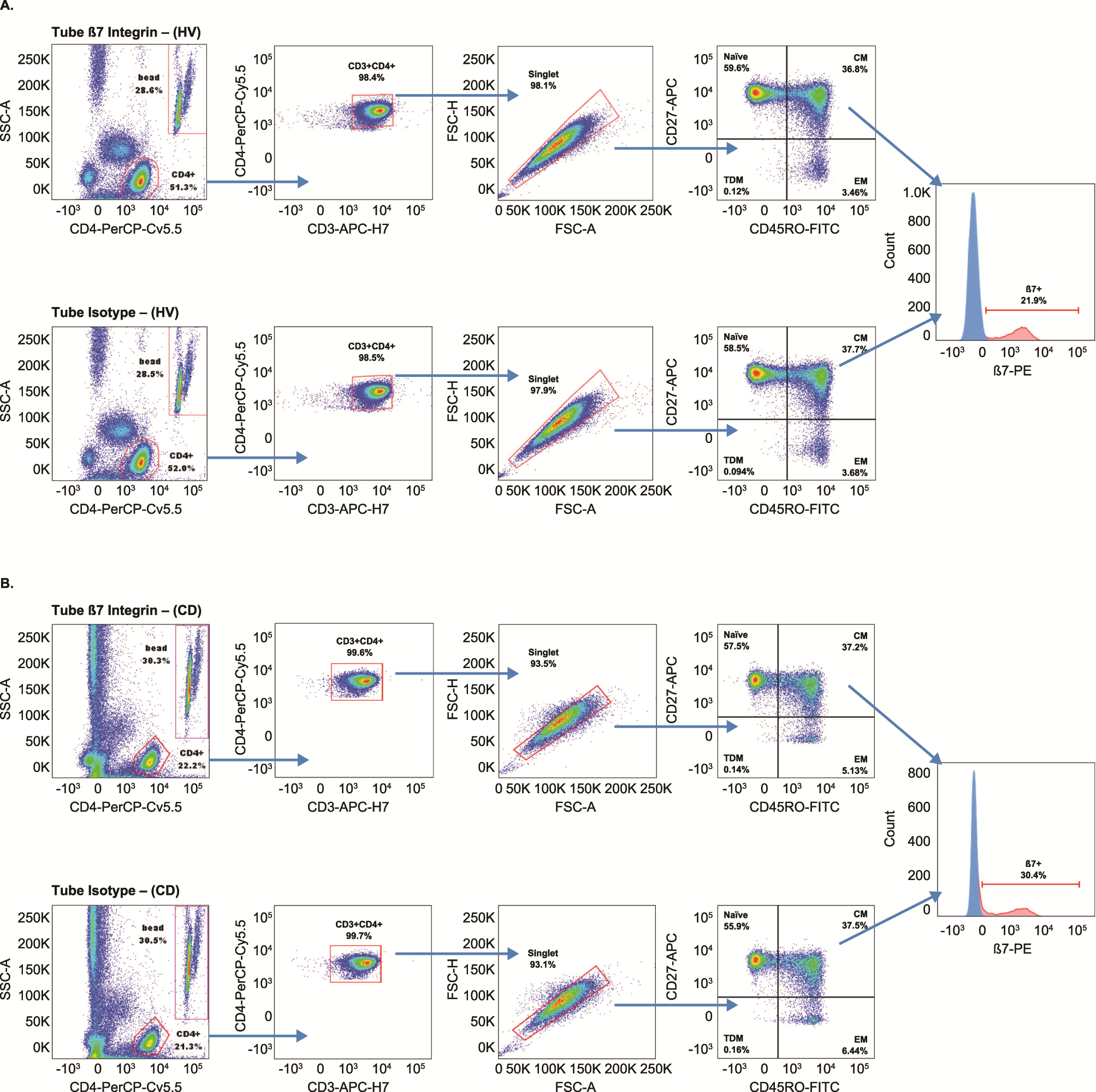

Supplement: Supplementary Figure S1 [file jjx121_suppl_supplementary_figure_s1.png]

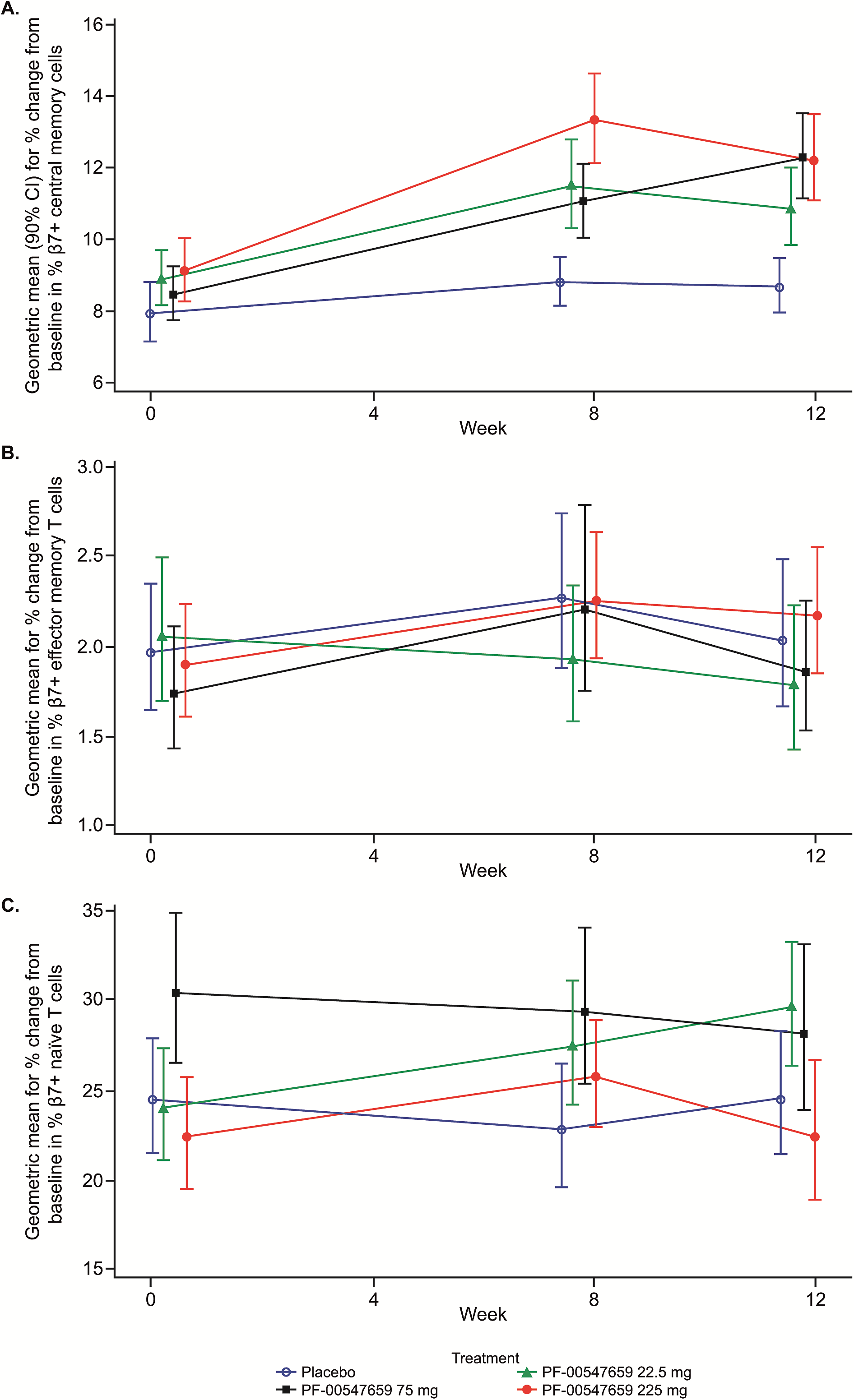

Supplement: Supplementary Figure S2 [file jjx121_suppl_supplementary_figure_s2.png]

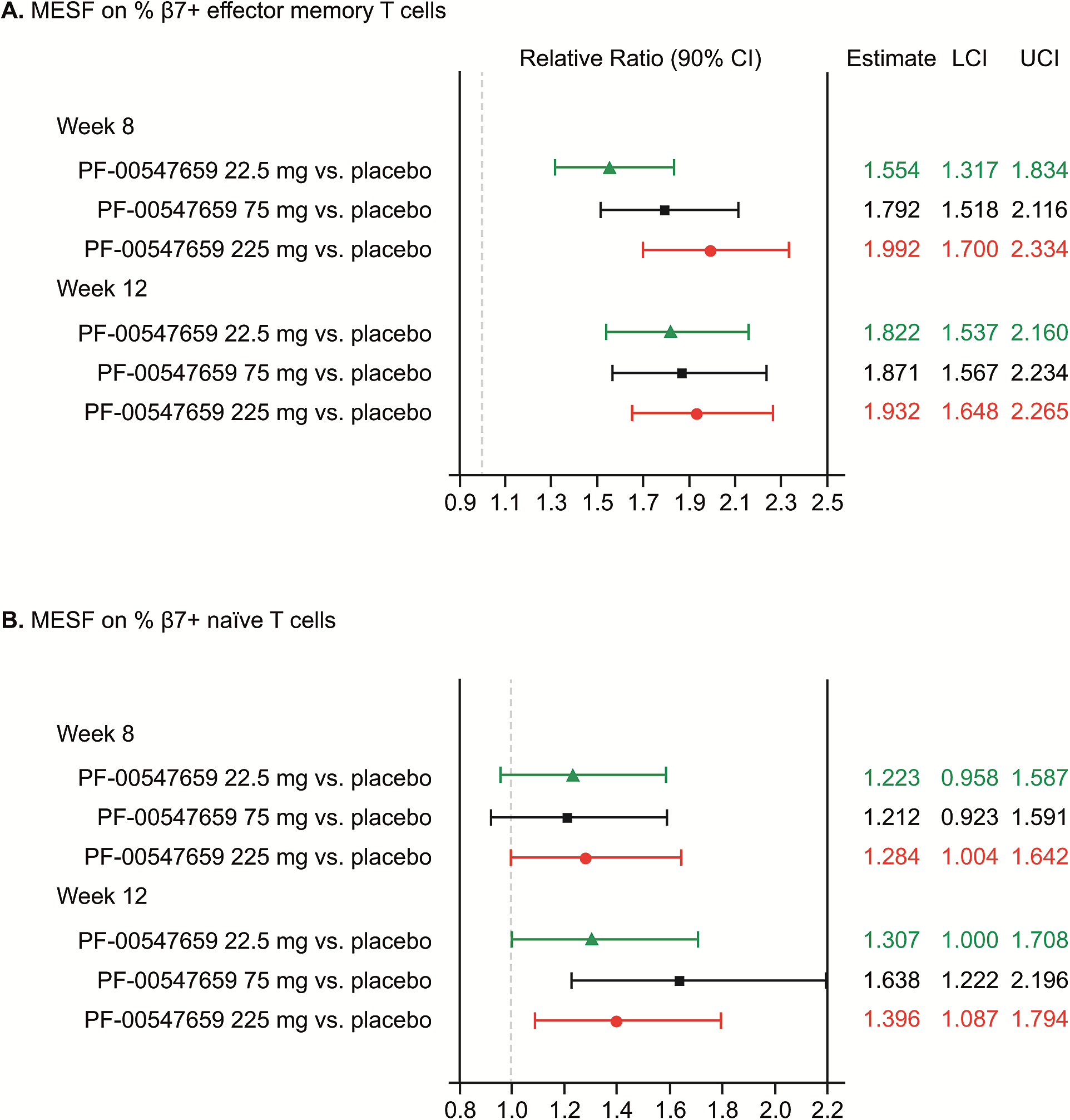

Supplement: Supplementary Figure S3 [file jjx121_suppl_supplementary_figure_s3.png]

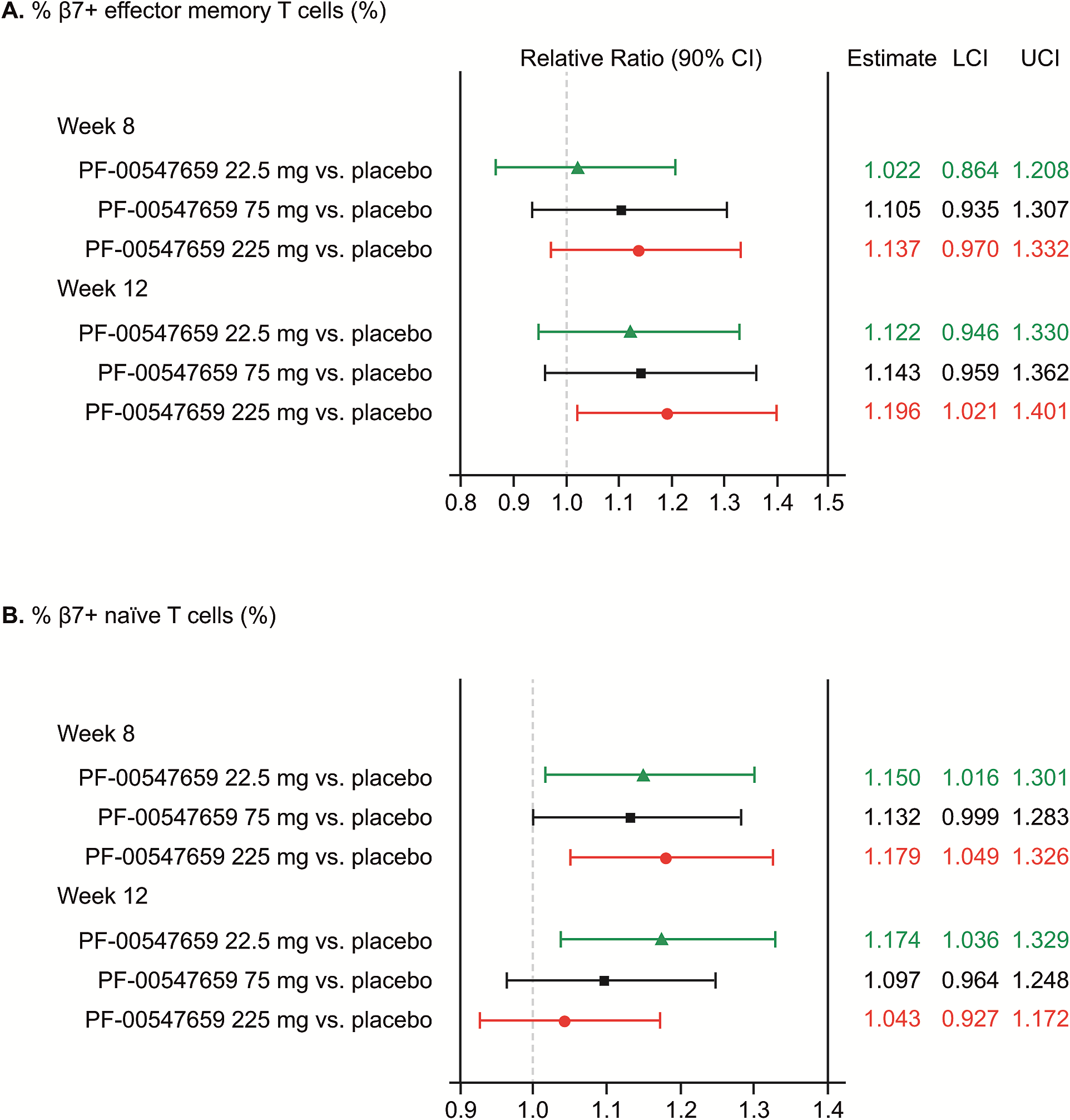

Supplement: Supplementary Figure S4 [file jjx121_suppl_supplementary_figure_s4.png]

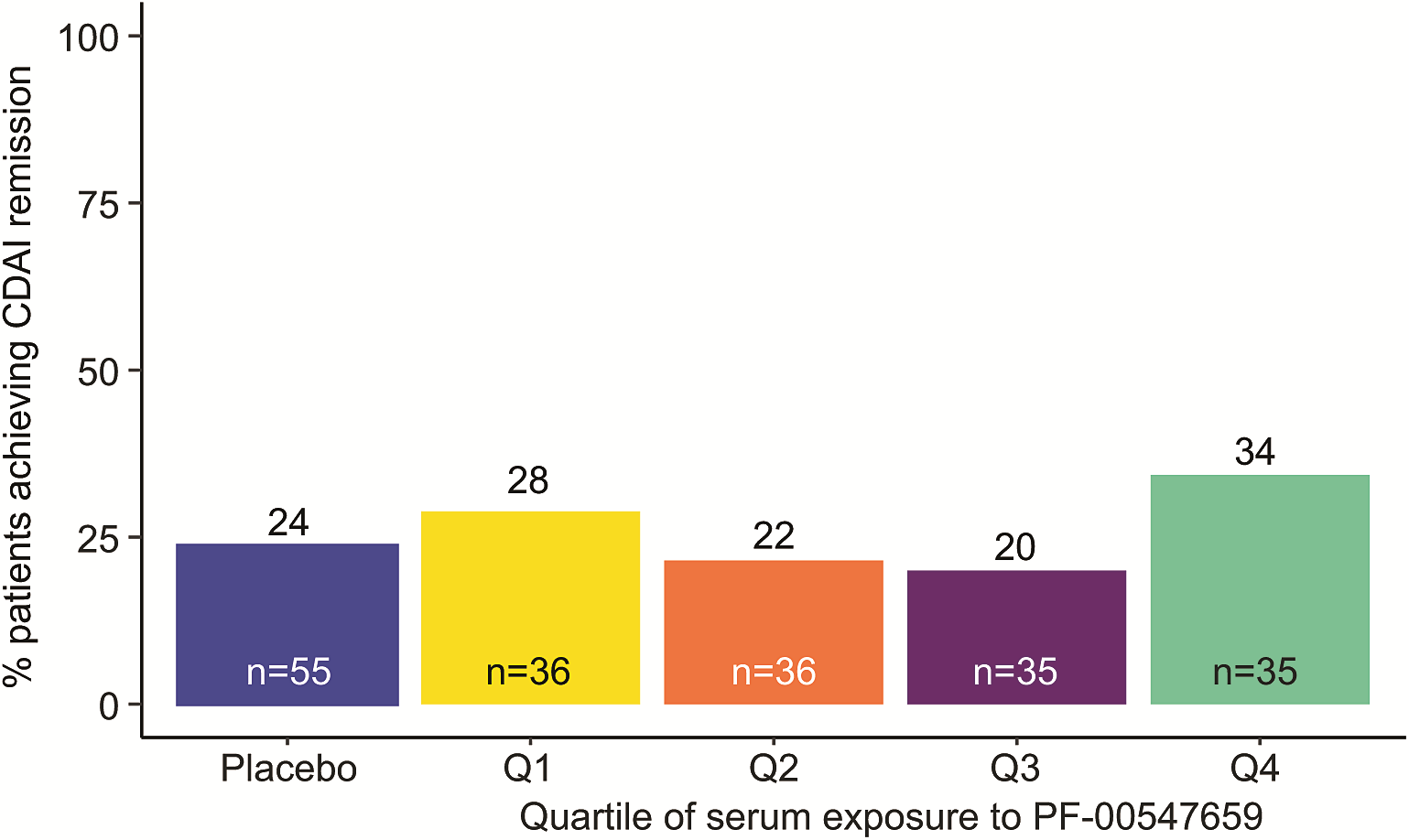

Supplement: Supplementary Figure S5 [file jjx121_suppl_supplementary_figure_s5.png]

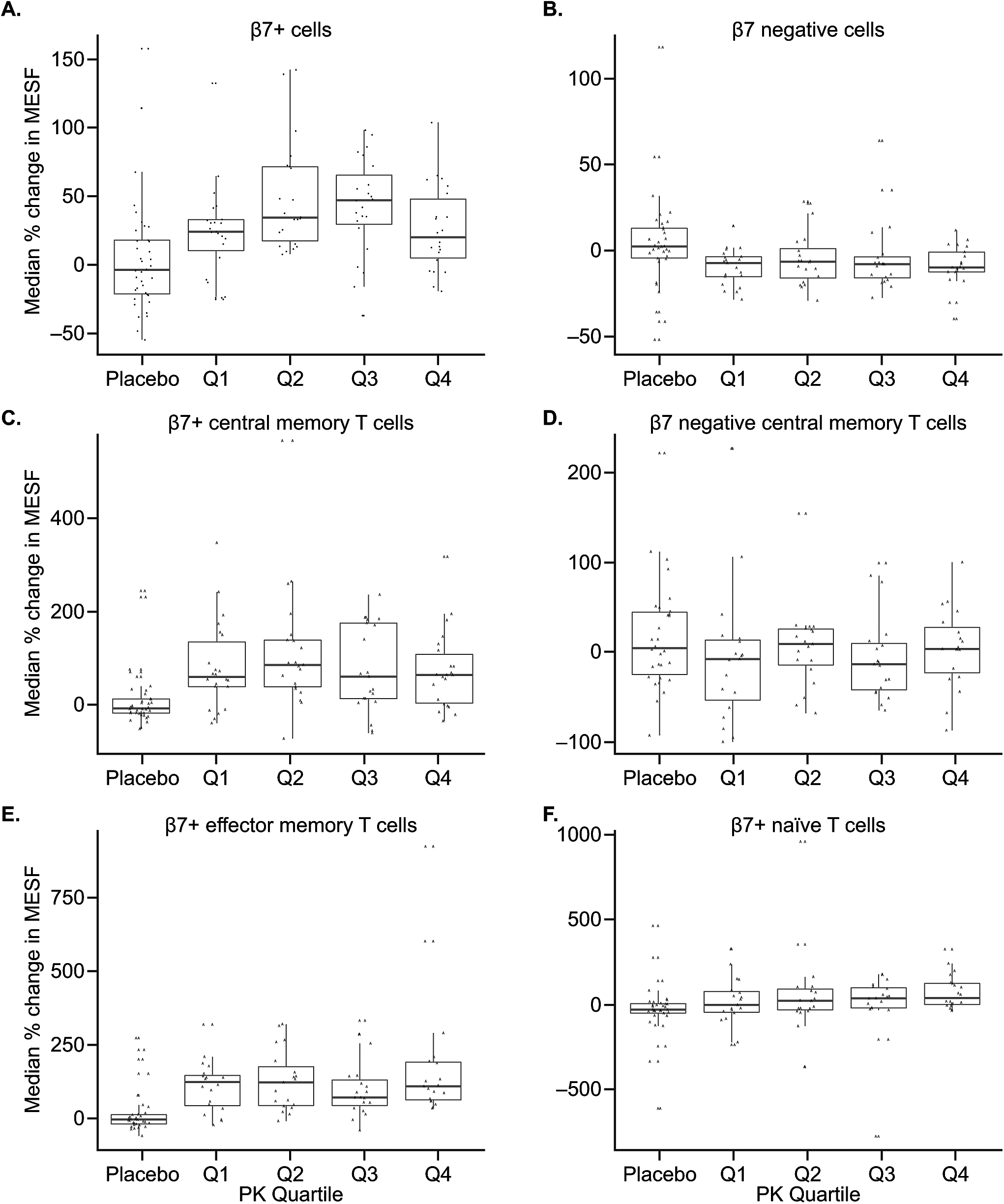

Supplement: Supplementary Figure S6 [file jjx121_suppl_supplementary_figure_s6.png]

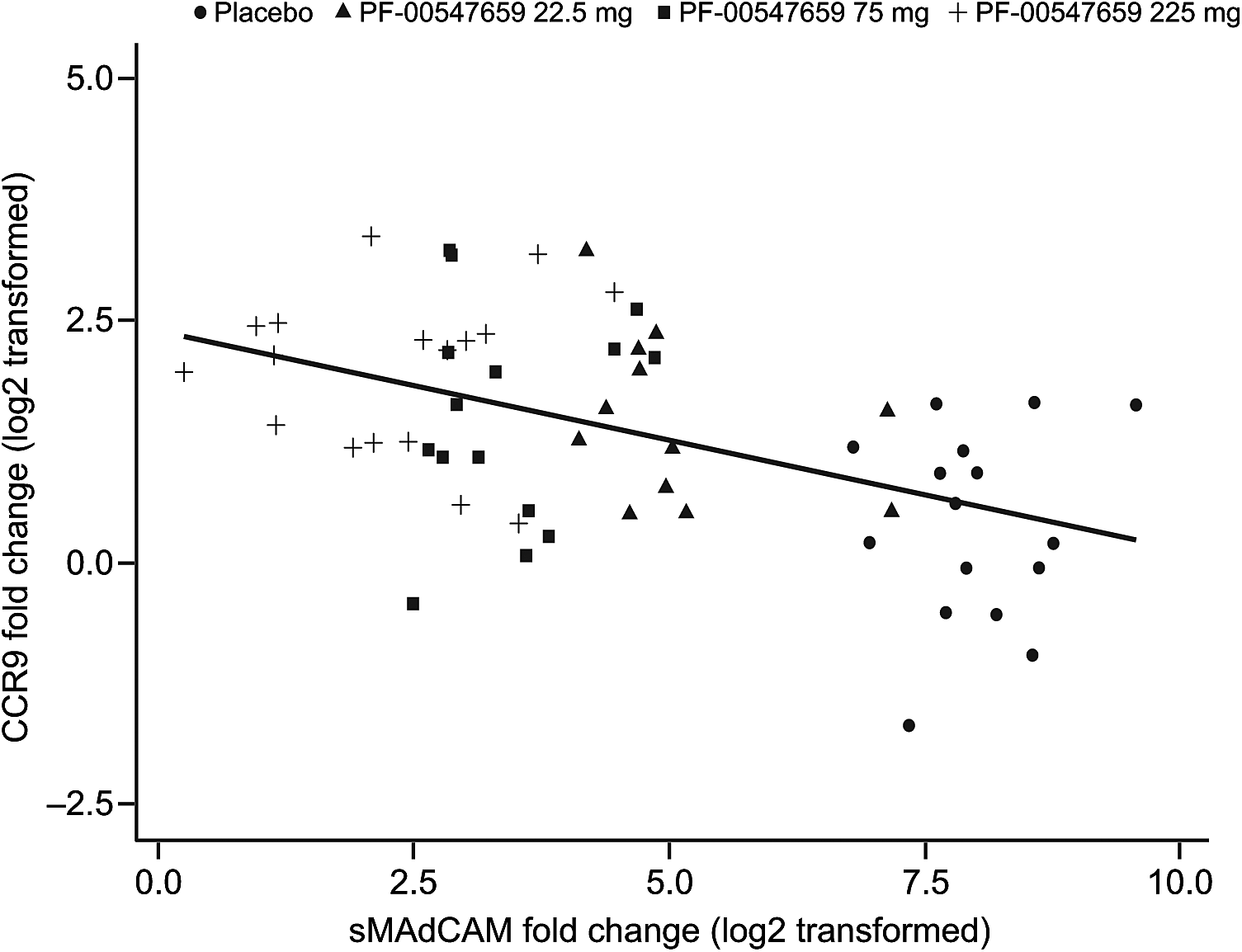

Supplement: Supplementary Figure S7 [file jjx121_suppl_supplementary_figure_s7.png]
