# Supplementary material for: Anti-MAdCAM Antibody Increases ß7+ T Cells and CCR9 Gene Expression in the Peripheral Blood of Patients With Crohn’s Disease
Source: J Crohns Colitis. 2017 Sep 7;12(1):77–86. doi: 10.1093/ecco-jcc/jjx121 (PMC5881777; doi:10.1093/ecco-jcc/jjx121)
Supplement: Supplementary Appendices [file jjx121_suppl_supplementary_appendices.docx]

**[[Supplementary Appendices]]**

**[[Supplementary Figure Legends]]**

**Supplementary Figure 1.** The gating strategy of the FACS β7 integrin assay. Each blood sample was analysed by a 2-tube (isotype control and β7 integrin), 5-color and lyze/no wash protocol. Raw data FCS files of isotype control and β7 samples from (A) a healthy volunteer (HV) and (B) a study patient with CD were analysed by Flowjo v10. As shown, identical hierarchical gating from CD4+ cells restriction to a quadrant plot of CD4+ naïve and memory subsets was applied to isotype and β7+ integrin in the HV and patient with CD. β7– and β7+ cells in each subset were set at 0.5% (range: 0.4% to 0.6%) of the CD3+CD4+ singlet of the isotype control. Overlapping histograms of the β7+ gate from the isotype (blue) and β7 (red) sample, expressed as % of parent, are shown on the right of panels (A) and (B). A bead gate assisted in deriving absolute β7 cell counts. The assay protocol and gating strategy were developed at Pfizer Lab and transferred to Quintiles Lab, which applied them to acquire and analyse all samples in this study. β7+ T cell frequency (of the CD3+CD4+ singlet), absolute count, and β7 molecule expression (calibrated by BD’s Quantibrite PE beads) parameters were reported in each CD4+ subset.

CM, central memory subset; EM, effector memory subset; TDM, terminally differentiated memory subset.

**Supplementary Figure 2.** Effect of PF-00547659 on (A) % β7+ central memory T cells; (B) % β7+ effector memory T cells; and (C) % β7+ naïve T cells in CD4+ cell subsets of patients with CD. β7 frequency is expressed as % of [CD3+CD4+singlet]. (See Supplementary Figure 1 for gating strategy.)

The error bars are geometric mean estimates (90% CI) over time for % change from baseline.

CI, confidence interval.

**Supplementary Figure 3.** The relative ratios (90% CI) of active treatment to placebo for MESF on (A) β7+ effector memory T cells to placebo; and (B) β7+ naïve T cells to placebo.

CI, confidence interval; LCI, lower confidence interval; MESF, molecules of equivalent soluble fluorochrome; UCI, upper confidence interval.

**Supplementary Figure 4.** The relative ratios (90% CI) of active treatment to placebo for (A) % β7+ effector memory T cells; and (B) % β7+ naïve T cells.

CI, confidence interval; LCI, lower confidence interval; UCI, upper confidence interval.

**Supplementary Figure 5.** Systemic exposure-response relationship between PF-00547659 levels (ng/ml) at Week 12 and CDAI clinical remission (CDAI < 150). Serum exposure to PF-00547659 is divided into quartiles: Q1 = 0 to 1.13 × 10^3^ ng/ml; Q2 = 1.13 × 10^3^ ng/ml to 5.20 × 10^3^ ng/ml; Q3 = 5.20 × 10^3^ ng/ml to 1.28 × 10^4^ ng/ml; Q4 = 1.28 × 10^4^ to 4.08 × 10^4^ ng/ml.

**Supplementary Figure 6.** Median % changes from baseline in MESF of β7+ cells [total T cells (A); central memory T cells (C); effector memory T cells (E); and naïve T cells (F)] and β7 negative cells [total T cells (B); and central memory T cells (D)] in the placebo group and in each quartile of PK levels of PF-00547659.

PK quartiles for PF-00547659: Q1 = 0 to 1.13 × 10^3^ ng/ml; Q2 = 1.13 × 10^3^ ng/ml to 5.20 × 10^3^ ng/ml; Q3 = 5.20 × 10^3^ ng/ml to 1.28 × 10^4^ ng/ml; Q4 = 1.28 × 10^4^ to 4.08 × 10^4^ ng/ml.

MESF, molecules of equivalent soluble fluorochrome; PK, pharmacokinetic.

**Supplementary Figure 7.** Relationship between CCR9 transcript levels from blood and serum sMAdCAM fold changes at Week 12. mRNA fold changes were calculated between Week 12 and baseline and were log2 transformed. An inverse linear relationship is observed between fold changes in CCR9 transcript and sMAdCAM levels. The four different symbols represent different treatment groups in the study: ● Placebo, ▲ PF-00547659 22.5 mg, ■ PF-00547659 75 mg, and + PF-00547659 225 mg.

sMAdCAM, soluble mucosal addressin cell adhesion molecule.

**[[Supplementary Appendices, Tables]]**

**Supplementary Table 1** Phenotype and population description of cells analysed by FACS

| **Phenotype** | **Population Description** |
| --- | --- |
| β7-CD45RO+CD27+[CD3+CD4+] % | β7-CD45RO+CD27+[CD3+CD4+] % (% β7 Negative Central memory T cells) |
| ABSOLUTE β7-CD45RO+CD27+[CD3+CD4+] | ABS β7-CD45RO+CD27+[CD3+CD4+] (ABS β7 Negative Central memory T cells) |
| MESF β7-CD45RO+CD27+[CD3+CD4+] | MESF on % β7 Negative (Central memory T cells) |
| CD4+CD45RO+CD27+β7 INTEGRIN+%(CD4+) | CD4+CD45RO+CD27+β7 INTEGRIN+%(CD4+) (% β7 Positive Central memory T cells) |
| CD4+CD45RO+CD27+β7 INTEGRIN+ABS | CD4+CD45RO+CD27+β7 INTEGRIN+ABS (ABS β7 Positive Central memory T cells) |
| CD4+CD45RO+CD27+β7 INTEGRIN+MESF | CD4+CD45RO+CD27+β7 INTEGRIN+MESF (MESF on % β7 Positive Central memory T cells) |
| β7+CD45RO-CD27+CD3+CD4+ % | β7+CD45RO-CD27+CD3+CD4+% (% β7 Positive Naïve T cells) |
| β7+CD45RO-CD27+CD3+CD4+ABS | β7+CD45RO-CD27+CD3+CD4+ABS (ABS β7 Positive Naïve T cells) |
| β7+CD45RO-CD27+CD3+CD4+MESF | β7+CD45RO-CD27+CD3+CD4+MESF (MESF on % β7 Positive Naïve T cells) |
| β7+CD45RO+CD27-CD3+CD4+ % | β7+CD45RO+CD27-(CD3+CD4+)% (% β7 Positive Effector Memory T cells) |
| β7+CD45RO+CD27-CD3+CD4+ABS | β7+CD45RO+CD27-(CD3+CD4+)ABS (ABS β7 Positive Effector Memory T cells) |
| β7+CD45RO+CD27-CD3+CD4+MESF | β7+CD45RO+CD27-(CD3+CD4+)MESF (MESF on % β7 Positive Effector memory T cells) |

FACS, fluorescence activated cell sorting; MESF, molecules of equivalent soluble fluorochrome.

**Supplementary Table 2** Results (*p* < 0.05) from pathway analyses of top 2000 genes from IPA, GeneGo, MSigDB, and other sources. A Z score > 2 indicates activation of disease/biological function (orange); a Z score < –2 indicates inhibition of disease/biological function (blue)

| **Diseases and biologic functions** | **Z Score** | | | |
| --- | --- | --- | --- | --- |
|  | **Placebo** | **PF-00547659** | | |
|  |  | **22.5 mg** | **75 mg** | **225 mg** |
| Cell movement | 1.6 | –2.4 | –3.1 | –2.6 |
| Migration of cells | 1.9 | –2.3 | –2.6 | –2.0 |
| Microtubule dynamics | 0 | –3.4 | 0 | –3.3 |
| Cell movement of neutrophils | 0 | –1.9 | –2.5 | –1.8 |
| Homing of leukocytes | 0 | –1.8 | –2.6 | –1.6 |
| Adhesion of immune cells | 0 | –0.5 | –2.6 | –2.5 |
| Homing of cells | 0 | 0 | –3.2 | –2.4 |
| Cell movement of granulocytes | 0 | –1.4 | –2.5 | –1.6 |
| Chemotaxis of myeloid cells | 0 | –2.0 | –3.1 | 0 |
| Chemotaxis of leukocytes | 0 | –2.2 | –2.9 | 0 |
| Chemotaxis of phagocytes | 0 | –2.1 | –2.9 | 0 |
| Chemotaxis of neutrophils | 0 | –1.9 | –3.1 | 0 |
| Chemotaxis of cells | 0 | –1.8 | –3.2 | 0 |
| Leukocyte migration | 0 | –1.1 | –2.5 | –1.3 |
| Adhesion of blood cells | 0 | –0.5 | –2.2 | –2.1 |
| Cell movement of phagocytes | 0 | –1.1 | –2.1 | –1.4 |
| Cell movement of leukocytes | 0 | –0.9 | –2.2 | –1.3 |
| Migration of neutrophils | 0 | 0 | –1.5 | –2.1 |
| Migration of granulocytes | 0 | 0 | –2.0 | –1.5 |
| Engulfment of cells | 0 | –1.7 | –3.7 | –3.0 |
| Endocytosis | 0 | –1.5 | –3.1 | –2.6 |
| Internalization of cells | 0 | 0 | –3.5 | –2.0 |
| Phagocytosis of cells | 0 | 0 | –2.9 | –1.5 |
| Phagocytosis of blood cells | 0 | 0 | –2.5 | –1.2 |
| Cell death | –0.3 | –1.5 | –2.9 | –0.2 |
| Cell death of myeloid cells | 0 | –1.5 | –3.1 | –1.6 |
| Cell death of immune cells | 0 | –1.5 | –2.4 | –1.1 |
| Cell death of phagocytes | 0 | –0.8 | –2.7 | 0 |
| Cell death of blood cells | 0 | 0 | –2.6 | –1.0 |
| Cell survival | 0 | 0 | –0.4 | –3.2 |
| Cell viability | 0 | 0 | –0.5 | –3.5 |
| Cell viability of blood cells | 0 | 0 | –1.6 | –2.3 |
| Degranulation of phagocytes | 0 | –2.4 | –2.5 | 0 |
| Degranulation of cells | 0 | –1.8 | –2.8 | –1.1 |
| Degranulation of mast cells | 0 | 0 | –2.0 | –1.5 |
| Synthesis of reactive oxygen species | 0 | –2.7 | –2.3 | –0.9 |
| Production of reactive oxygen species | 0 | –2.6 | 0 | –1.1 |
| Cytotoxicity | 0 | 0 | –3.3 | –1.4 |
| Cytotoxicity of cells | 0 | 0 | –3.2 | –2.0 |
| Cytotoxicity of leukocytes | 0 | 0.6 | –3.0 | –1.5 |
| Cytotoxicity of lymphocytes | 0 | 0 | –2.5 | –1.3 |
| Synthesis of eicosanoid | 0 | –1.9 | –2.6 | 0 |
| Synthesis of lipid | 0 | 0 | –1.9 | –3.3 |
| Aggregation of cells | 0 | –1.7 | 0 | –2.4 |
| Apoptosis of leukaemia cell lines | 0 | –0.9 | –2.8 | 0 |
| Apoptosis of tumour cell lines | 0 | –2.1 | –1.8 | 1.1 |
| Arthritis | 0 | –2.1 | –1.5 | 0.1 |
| Bacterial infections | 0 | 1.4 | 3.1 | 0.1 |
| Binding of cells | 0 | 1.0 | –2.7 | –1.5 |
| Binding of neutrophils | 0 |  | –1.6 | –2.4 |
| Cancer | –0.3 | –2.1 | –2.5 | 0 |
| Damage of liver | 0 | 0 | –1.5 | –2.1 |
| Development of connective tissue | 0 | 0 | –1.8 | –2.3 |
| Development of haematopoietic progenitor cell | 0 | 0 | 1.9 | 2.3 |
| Development of haematopoietic system | 0 | 0 | 2.5 | 1.6 |
| Fatty acid metabolism | 0 | –2.3 | –1.2 | 0 |
| Formation of leukocytes | 0 | 0 | 1.7 | 2.1 |
| HIV infection | 0 | 0 | –1.4 | –3.7 |
| Hypersensitive reaction | 0 | 0 | –2.2 | –1.3 |
| Immune response of cells | 0 | –0.7 | –2.9 | –0.7 |
| Immune response of tumour cell lines | 0 | –0.1 | –2.2 | –1.2 |
| Infection by RNA virus | 0 | 0 | –1.7 | –3.8 |
| Infection of cells | 0 | 0 | –0.9 | –3.6 |
| Infection of tumour cell lines | 0 | 0 | –2.7 | –3.1 |
| Inflammatory response | 0 | –2.5 | –2.3 | –1.9 |
| Invasion of cancer cells | 0 | –1.4 | 0 | –2.2 |
| Invasion of epithelial tissue | 0 | –1.0 | 0 | –2.6 |
| Killing of bacteria | 0 | –2.8 | –2.4 | 0 |
| Necrosis | –0.1 | –1.1 | –2.3 | 0.5 |
| Orientation of cells | 0 | 0 | –2.5 | –2.2 |
| Polarization of cells | 0 | 0 | –2.4 | –2.0 |
| Rheumatic disease | 0 | –2.1 | –1.5 | 0.4 |
| Seizure disorder | 0 | 0 | 1.8 | 2.4 |
| Toxicity of cells | 0 | 0 | –3.1 | –1.8 |
| Viral infection | 0 | 0 | –1.7 | –4.4 |

**[[Supplementary Appendices]]**

**Appendix 1**

**Determination of soluble MAdCAM-1 in human serum**

***Procedures:*** A 100-μl aliquot of each sample was combined with streptavidin-coated magnetic beads and biotinylated PF-00547659. Reagent concentrations and incubation conditions were carefully optimized to minimize interference with drug-target binding. Following a washing procedure, captured soluble mucosal addressin cell adhesion molecule (sMAdCAM) was acid-eluted from the beads, and a stable isotope labelled (SIL) peptide standard added before reduction, alkylation, and overnight digestion. The target MAdCAM sequence GLDTSLGAVQSDTGR and its SIL peptide standard were separated by online anti-peptide antibody immunoaffinity linked to nanoflow reverse phase chromatography, as in a previously described procedure,^1^ and detected by mass spectrometry. The high specificity of this assay was conferred by the two immunoaffinity enrichment steps, at protein and tryptic peptide level, together with detection of the proteotypic peptide GLDTSLGAVQSDTGR that is unique to human MAdCAM-1. A human MAdCAM-1 Fc was used as a reference standard over a calibration range from 0.5 to 512.0 pM.

***Validation:*** The assay was validated in three independent batch runs using multiple validation standards at multiple concentrations, with six replicates in each run. Intra- and inter-batch coefficients of variation (% CV) and relative errors (% RE) were typically less than 10%, and less than 15% in all cases.

**Analytical method for determination of sMAdCAM-1 in human serum**

sMAdCAM was measured from serum samples using an immunoaffinity liquid chromatography tandem mass spectrometry (IA-LC-MS/MS) assay. A 100-μl aliquot of each serum study, calibrant, or quality control sample was combined with 10 μl of washed streptavidin-coated magnetic beads (DynaBeads SA T1, Invitrogen Life Technologies, Carlsbad, CA) and 10 μl biotinylated PF-00547659 (10 μg/ml) before dilution with phosphate buffered saline (PBS) to 800 μl in a 1-ml Protein LoBind^®^ Deepwell Plate with 96 wells (Eppendorf AG, Hamburg, Germany). Following incubation at room temperature for 1 hour while shaking, the magnetic beads were removed from the samples using the Microlab Star^®^ automated liquid handling system (Hamilton, Bonaduz, Switzerland), as described in previously published methodology.^2^ The beads were subsequently washed twice with 280 μl of 0.05% Tween-20 PBS buffer followed by one wash with 280 μl PBS. Captured sMAdCAM was eluted from the beads with two 70-μl aliquots of 25 mM HCl and collected in a fresh 1-ml Protein LoBind Deepwell Plate with 96 wells containing 15 μl of 2 M Tris–HCl, pH 8.0. A 15-μl aliquot of 1 fmol/μl winged SIL peptide standard (ASVQWRGLDTSLGAVQSDTG***R***SVLTVR; ***R*** = 10 Da mass shift, peptide ID OR286536, peptide purity > 97; Thermo Biopolymers, Ulm, Germany) in 0.1 μg/ml glucagon in PBS was added to each sample. Following the addition of 35 μl of 10 M urea and 10 μl of 40 mM DTT, the samples were incubated at 56°C for 30 minutes. Subsequently, the plate was left to cool to room temperature for 10 minutes, before 10 μl of 80 mM iodoacetamide was added and the samples incubated in the dark at room temperature for 30 minutes. Samples were then digested overnight at 37°C using 10 μl of 100 μg/ml of trypsin (Promega Corporation, Madison, WI). Detailed conditions for online anti-peptide antibody immunoaffinity separation linked to nanoflow reverse phase chromatography have been described previously.^1^ Briefly, a Dionex UltiMate^®^ 3000 system was configured with a WPS-3000 Autosampler (Thermo Scientific, Sunnyvale, CA), two micropumps, and one pump capable of nanoflow rates. A 3 x 2.1 mm column containing a polyclonal rabbit anti-peptide antibody directed against the target MAdCAM sequence GLDTSLGAVQSDTGR was held at 15°C. A PepMap300 C18 pre-column (5 × 0.3 mm, 5 μm, 300 Å, Dionex) and a PepMap300 Acclaim C18 column (15 cm × 75 μm, 5 μm, 300 Å, Dionex) were held at 70°C. The total chromatography duty cycle (including injection) was 10 minutes. The nanoflow column effluent was introduced into a Vantage triple quadrupole mass spectrometer (Thermo Scientific) using a TriVersa NanoMate with ESI chip (Advion, Ithaca, NY) under the following conditions: LC coupling spray voltage, 1.7kV; capillary temperature, 250°C; and collision gas pressure, 1.7 mTorr. The following selected reaction monitoring (SRM) transitions were recorded for the doubly charged native peptide Q1 738.9 [M+2H]^2+^ to Q3 890.4 (y_9_^1+^) and SIL peptide Q1 743.8 [M+2H]^2+^ to Q3 900.4 (y_9_^1+^). The collision energy was 26 eV and the dwell time was 50 msec.

The peak area ratios of sMAdCAM and SIL peptide were determined using LC-QUAN™ Version 2.6 (Thermo Fisher Scientific) and sMAdCAM concentrations were calculated by Watson™ version 7.3 (Thermo Fisher Scientific). The high measurement specificity of this assay was conferred by the two immunoaffinity enrichment steps, at protein and tryptic peptide level, together with the detection of proteotypic peptide GLDTSLGAVQSDTGR, which is unique to human MAdCAM-1. Human MAdCAM-1 Fc (R&D Systems, Minneapolis, MN, 6056-MC) in 5% bovine serum albumin (BSA)/PBS was used as a reference standard over calibration range from 0.5 to 512 pM using a linear curve fit applying a 1/x weighting. The assay was validated in three independent batch runs using the following validation standards (VS), each at six replicates in each run, using the specified sMAdCAM concentrations: endogenous MAdCAM in human serum pool (END); 1.33-fold dilution of END (VS Medium 1), 20-fold dilution of END (VS Medium 2), 200-fold dilution of END (VS Low), 100 pM MAdCAM-1 Fc spike to END (VS High), 0.5 pM and 512 pM preparation of sMAdCAM Fc in 5% BSA/PBS (VS Lower Limit Of Quantification and VS Upper Limit Of Quantification).

**Flow cytometric assays**

***Reagents and supplies:*** Conjugated monoclonal antibodies against CD138 (FITX, Cat. No. 552723), CD10 (PE, Cat. No. 340921), CD45 PerCP (Cy5.5, Cat. No. 340437), CD19 (APC, Cat. No. 340437), CD38 (Pe-Cy7, Cat. No. 335790), CD27 (V450, Cat. No. 560448), CD3 (APC-H7, Cat. No. 641406), CD45RO (FITC, UCHL1 Cat. No. 555492), β7 integrin (PE, FIB504 Cat. No. 555945), CD4 PerCP Cy5.5 (Sk3, Cat. No. 332772), CD27 APC (L128, Cat. No. 337169), and CD3 APC-H7 (SK7, Cat. No. 641415) were used in the assays (Becton Dickinson, San Jose, CA). An isotype control rat IgG2a Isotype (PE, R35-95 Cat. No. 555844; Becton Dickinson) and Beckman Coulter Stem kit (IM3630; Beckman Coulter, Inc, Miami, FL) were also utilized.

***β7 integrin analysis:*** Aliquots (100 µl) of whole blood were incubated with 30 μl of the antibody cocktail (CD45RO-FITC, β7-integrin or rat IgG2a isotype control-PE, CD4-PerCPCy5.5, CD27-APC, and CD3-APC-H7) in BD TruCount***^®^*** tubes (Cat. No. 340334; Becton Dickinson) at room temperature for 30 minutes. One ml of 1X lysing solution (BD Pharm Lyse, Cat. No. 555899; BD Bioscience, San Jose, CA) was added to each tube, shaken by hand, and incubated for 30 minutes at room temperature in the dark for 30 minutes. Lysed blood was subsequently analysed using the BD FACSCanto II^®^ flow cytometer (BD Biosciences) within 2 hours of preparation. The FACSCanto II was set to acquire most of the sample per tube to ensure maximum events possible, and thus increase precision.

To determine cell surface β7 levels, the frequency and absolute number of β7+ cells in CD4+CD27+CD45RO+ (central memory T cells), CD4+CD27-CD45RO+ (effector memory T cells), and CD4+CD27+CD45RO– (naïve T cells) populations were measured, and the unit of molecules of equivalent soluble fluorochrome (MESF; i.e., the unit measure of β7 protein expression on T cells) was calculated. Percentage of β7+ data was reported as the percentage of CD4+ cells that also expressed β7; absolute cell counts were derived using BD TruCount^®^ beads (Becton Dickinson).

**Appendix 2**

**Gene expression profiling analysis**

***Blood sample collection, RNA extraction, globin mRNA depletion, and RNAseq:*** Peripheral venous blood samples were collected from patients at baseline and Week 12 into PAXgene Blood RNA tubes (PreAnalytiX GmbH, BD Biosciences, Mississauga, ON, Canada) according to the manufacturer’s protocol and stored at –80°C until processing. RNA extraction and quality control were performed by LabCorp (Seattle, WA). The yield and quality of the isolated RNA was assessed using Ribogreen (custom method using Life Technologies R11490) and Agilent 2100 Bioanalyzer (Agilent Technologies, Santa Clara, CA), respectively. Prior to cDNA library construction, globin reduction was performed by BGI (Tai Po, Hong Kong) on 1.5 mg of RNA using the GlobinClear kit (cat# AM1980; Life Technologies, Carlsbad, CA). For samples with a mass ≤ 500 ng or a RIN ≤ 6 and mass ≤ 1 µg, library construction proceeded without globin reduction.

***cDNA library construction and sequencing:*** cDNA libraries were prepared at BGI from 300 ng of RNA with a TruSeq stranded mRNA library prep Kit (cat# RS-122-2101, Illumina, San Diego, CA) according to the manufacturer’s protocol. The resulting libraries were sequenced on a HiSeq 2000 sequencer (Illumina) using a paired-end run (2×100 bases). A minimum of 40 M reads were generated from each library. For samples in which globin reduction was not applied, a minimum of 100 M reads were generated.

***Data processing and differential gene expression analysis:*** The clean raw sequence reads were mapped to human genome reference Hg19 using STAR^3^ and then counts of uniquely mapped reads that overlapped with a flattened bed file of exons were summarized on the gene level as raw expression values by BEDTools.^4^ EdgeR^1^ was then used to normalize the counts and to perform the differential gene expression analysis. A batch script was written to download, unzip, and merge the hg19 genome sequences in FASTA format from the UCSC website (ftp://hgdownload.cse.ucsc.edu/goldenPath/hg19/chromosomes). The comprehensive gene annotation (GENCODE v18) in GTF format was downloaded from http://www.gencodegenes.org/releases/18.html.

The clean raw sequence reads in FASTQ format were first mapped to the human reference genome version hg19 using STAR v2.3.0e.^5^ The uniquely mapped reads were counted towards individual genes by the program BEDTools.^6^ At first, a flattened bed file was generated based on GENCODE exon definition by merging overlapped exons for a gene into fused intervals, which can belong to more than one exon. Reads falling into these disjoined intervals were counted using BEDTools and then summarized into the gene level.

The parameters for BEDTools v2.18.2 run were “-wo -split -bed -abam sample.bam -b hg19.flatten.bed.” To reduce false positives in the differential analysis, genes with less than 2 reads in more than 80% samples and an average of read counts less than 3 across all samples were treated as not expressed, and thus omitted from downstream data analysis. The differential analysis of genes changing from baseline to Week 12 was carried out by R packages EdgeR^7^ based on the gene counts table generated in the previous step.

***Pathway analysis:*** Ingenuity Pathway Analysis (Qiagen), MetaCore (Thomson Reuters), and Pfizer’s internal Causal Reasoning Engine Knowledgebase^8^ were used to gain insights into 1) the mechanism of action of the anti-MAdCAM-1 antibody PF-00547659 with a focus on T effector (Teff) cells, T central memory (Tcm) cells, and T regulatory (TReg) cells using 2000 genes associated with treatment response in any dose group; and 2) the dose-dependent modulation of pathways using differentially expressed genes in the groups receiving placebo or 22.5 mg, 75 mg, or 225 mg of PF-00547659 at 12 weeks versus baseline. *P*-values were calculated using a right-tailed Fisher’s exact test to determine statistically significant over-representation of genes in pathways, networks, and functional processes. Z-scores as reported by Ingenuity Pathway Analysis predict the activation (positive Z-scores) or repression (negative Z-scores) of pathways/networks/functional processes based on the observed gene expression changes and the underlying knowledge in the database. A *p*-value of < 0.05 and a Z-score of ≤ –2 or ≥ 2 were established as significance cutoffs for identified pathways/networks/functional processes.

**Appendix 3**

**Statistical analysis of gene expression data**

The following three major analyses were performed:

1. Identification of genes that were differentially expressed among treatment groups. Linear models were employed with change of gene expression as response variable and age, baseline gene expression, gender, anti-tumour necrosis factor (TNF), and treatment as independent variables.
2. Identification of genes whose baseline expressions can predict the achievement of the primary clinical endpoint, CDAI70 [≥ 70-point decrease in CDAI from baseline]. Logistic regression analyses were employed with CDAI70 as response variable and age, baseline gene expression, baseline CDAI score, gender, anti-TNF, treatment, and gene-by-treatment interaction as independent variables.
3. Identification of genes whose changes of expression between baseline and Week 12 was associated with change of sMAdCAM or α-β7. Spearman rank correlations were calculated for each gene.

In each analysis, Q values were calculated to control false discovery rate.^9^ A biomarker with Q value < 0.1 will be considered significant. Statistical computing software R 3.1.0 and packages including Q value were used in the statistical analyses.

**References**

1. Palandra J, Finelli A, Zhu M, Masferrer J, Neubert H. Highly specific and sensitive measurements of human and monkey interleukin 21 using sequential protein and tryptic peptide immunoaffinity LC-MS/MS. *Anal Chem*. 2013;85:5522-5529.
2. Neubert H, Muirhead D, Kabir M, Grace C, Cleton A, Arends R. Sequential protein and peptide immunoaffinity capture for mass spectrometry-based quantification of total human beta-nerve growth factor. *Anal Chem*. 2013;85:1719-1726.
3. Sun H, Liu J, Zheng Y, Pan Y, Zhang K, Chen J. Distinct chemokine signaling regulates integrin ligand specificity to dictate tissue-specific lymphocyte homing. *Dev Cell.* 2014;30:61-70.
4. Singh H, Grewal N, Arora E, Kumar H, Kakkar AK. Vedolizumab: a novel anti-integrin drug for treatment of inflammatory bowel disease. *J Nat Sci Biol Med*. 2016;7:4-9.
5. Dobin A, Davis CA, Schlesinger F, et al. STAR: ultrafast universal RNA-seq aligner. *Bioinformatics*. 2013;29:15-21.
6. Quinlan AR, Hall IM. BEDTools: a flexible suite of utilities for comparing genomic features. *Bioinformatics*. 2010;26:841-842.
7. Robinson MD, McCarthy DJ, Smyth GK. edgeR: a Bioconductor package for differential expression analysis of digital gene expression data. *Bioinformatics*. 2010;26:139-140.
8. Chindelevitch L, Ziemek D, Enayetallah A, et al. Causal reasoning on biological networks: interpreting transcriptional changes. *Bioinformatics*. 2012;28:1114-1121.
9. Dalmasso C. Estimation of the false recovery rate. R package version 1.26.0. 2007 [updated 2007; cited 2015 December 9, 2015]; R package version 1.26.0: Available from: <http://citeseerx.ist.psu.edu/viewdoc/download?doi=10.1.1.304.829&rep=rep1&type=pdf>.
